# Establishment of a risk score model for bladder urothelial carcinoma based on energy metabolism‐related genes and their relationships with immune infiltration

**DOI:** 10.1002/2211-5463.13580

**Published:** 2023-03-05

**Authors:** Caihong Huang, Yexin Li, Qiang Ling, Chunmeng Wei, Bo Fang, Xingning Mao, Rirong Yang, LuLu Zhang, Shengzhu Huang, Jiwen Cheng, Naikai Liao, Fubo Wang, Linjian Mo, Zengnan Mo, Longman Li

**Affiliations:** ^1^ Center for Genomic and Personalized Medicine, Guangxi Key Laboratory for Genomic and Personalized Medicine, Guangxi Collaborative Innovation Center for Genomic and Personalized Medicine Guangxi Medical University Nanning China; ^2^ Department of Urology Institute of Urology and Nephrology, First Affiliated Hospital of Guangxi Medical University Nanning China; ^3^ School of Public Health Guangxi Medical University Nanning China

**Keywords:** bladder urothelial carcinoma, energy metabolism, immune, prognosis, risk score model

## Abstract

Bladder urothelial carcinoma (BLCA) is a common malignant tumor of the human urinary system, and a large proportion of BLCA patients have a poor prognosis. Therefore, there is an urgent need to find more efficient and sensitive biomarkers for the prognosis of BLCA patients in clinical practice. RNA sequencing (RNA‐seq) data and clinical information were obtained from The Cancer Genome Atlas, and 584 energy metabolism‐related genes (EMRGs) were obtained from the Reactome pathway database. Cox regression analysis and least absolute shrinkage and selection operator analysis were applied to assess prognostic genes and build a risk score model. The estimate and cibersort algorithms were used to explore the immune microenvironment, immune infiltration, and checkpoints in BLCA patients. Furthermore, we used the Human Protein Atlas database and our single‐cell RNA‐seq datasets of BLCA patients to verify the expression of 13 EMRGs at the protein and single‐cell levels. We constructed a risk score model; the area under the curve of the model at 5 years was 0.792. The risk score was significantly correlated with the immune markers M0 macrophages, M2 macrophages, CD8 T cells, follicular helper T cells, regulatory T cells, and dendritic activating cells. Furthermore, eight immune checkpoint genes were significantly upregulated in the high‐risk group. The risk score model can accurately predict the prognosis of BLCA patients and has clinical application value. In addition, according to the differences in immune infiltration and checkpoints, BLCA patients with the most significant benefit can be selected for immune checkpoint inhibitor therapy.

## Graphical abstract

Reprogramming of energy metabolism is closely related to cancer. In this study, we constructed a prognostic model of bladder urothelial carcinoma based on energy metabolism‐related genes and explored the differences in the immune microenvironment and immune checkpoints between high‐risk and low‐risk groups, which will help to accurately select patients who are more likely to benefit from immunotherapy.

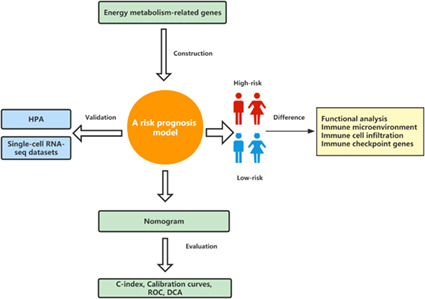

**Abbreviations**
AUC: area under the curve
BLCA: bladder urothelial carcinoma
CI: confidence interval
*C*‐index: index of concordance
DCA: decision curve analysis
EMRGs: energy metabolism‐related genes
FC: fold‐change
FDR: false discovery rate
GO: Gene Ontology
HPA: Human Protein Atlas
HR: hazard ratio
KEGG: Kyoto Encyclopedia of Genes and Genomes
LASSO: least absolute shrinkage and selection operator
OS: overall survival
RNA‐seq: RNA sequencing
ROC: time‐dependent receiver operating characteristic
TCGA: The Cancer Genome Atlas

Bladder urothelial carcinoma (BLCA) is a frequent malignant tumor of the human genitourinary system and is estimated to be the tenth most common cancer worldwide by 2020, with approximately 573 000 new instances and 213 000 deaths [1]. In recent years, although the surgical treatment, radiotherapy, adjuvant chemotherapy, and immunotherapy of BLCA have made significant progress [2, 3, 4], there is still a significant proportion of BLCA patients with poor prognosis [5]. For example, the 5‐year overall survival (OS) rate used to be much < 50% in patients with muscular infiltrating bladder [6]. The lack of high‐quality prognostic markers is one of the main reasons for the poor prognosis of BLCA [7]. Therefore, there is an urgent need to find more efficient and sensitive biomarkers for personalized treatment and better prognosis of BLCA patients.

Energy metabolism has long been considered to be an essential hallmark of most cancers, providing reductive oxidative stability and important macromolecular biosynthesis for tumor cell growth, migration, and invasion through the production of adenosine triphosphate [8]. Multiple energy metabolic pathways, such as aerobic glycolysis, oxidative phosphorylation, pyruvate metabolism, and the tricarboxylic acid cycle, are engaged in the prevalence and progression of human malignant tumors [8]. Tumor cells have dynamic metabolic heterogeneity to adapt to the complicated and changeable tumor microenvironment [9, 10]. Many tumor cells prefer aerobic glycolysis to generate energy, while others show an oxidative phosphorylation metabolic phenotype [11, 12]. Aerobic glycolysis is a common form of metabolic reprogramming in which tumor cells turn glucose into lactic acid in the presence of oxygen, creating an acidic environment [13]. It has been observed that BLCA patients exhibit a range of metabolic changes, together with expanded aerobic glycolysis, extended pyruvate metabolism, and imbalance of glycogen and oxidative metabolism, which together promote the survival, proliferation, and migration of tumor cells [14]. Zhang *et al*. [15] divided patients into four metabolic subtypes (mixed, cholesterol, glycolytic, and quiescent) based on glycolysis and cholesterol production genes, and the mixed subtypes were significantly associated with a poorer prognosis than the stationary subtypes. Zhu *et al*. [16] developed a reliable prognostic model based on 11 genes related to lipid metabolism, which can be adopted to predict prognosis and response to immunotherapy in BLCA patients. It follows that tumor energy metabolism might be of great significance for the prognosis and treatment of BLCA patients. Therefore, it would be a promising strategy to discover more sensitive biomarkers from the perspective of energy metabolism.

Here, we developed a risk score model based on 13 energy metabolism‐related genes (EMRGs) for predicting OS in patients with BLCA, employing BLCA transcriptome data and clinical information from The Cancer Genome Atlas (TCGA) database. Differences in the immune microenvironment, immune infiltration, immune checkpoint, and key biological functions among patients with different prognostic risks were assessed. Additionally, we verified the expression of EMRGs from the risk score model combined with the Human Protein Atlas (HPA) database and our single‐cell RNA sequencing (RNA‐seq) dataset of BLCA patients at the protein and single‐cell levels.

## Materials and methods

### Patients and preprocessing

The RNA‐seq data and clinical information of BLCA patients were downloaded from the TCGA database (https://portal.gdc.cancer.gov), and a total of 414 BLCA samples were included. As Yu *et al*. described previously [17], the BLCA patient dataset from TCGA was cleaned as follows. First, we deleted research subjects with no follow‐up data (including survival status, follow‐up time, and survival time). Second, genes whose mean expression level was less than or equal to zero in all samples were excluded. Third, gene annotation was performed using the Ensemble database. Finally, we selected the mean value as the expression level of the gene in the presence of multiple identical gene symbols. Eventually, 396 BLCA samples were enrolled.

From August 2019 to January 2020, we collected 10 tumor tissue samples from eight patients with BLCA initially diagnosed by surgery and pathological examination from the First Affiliated Hospital of Guangxi Medical University and the Guangxi Medical University Cancer Hospital. The research was approved by the Medical Ethics Committee of the First Affiliated Hospital of Guangxi Medical University (approval no. 2017YFO0908000), and written informed consent was obtained from all participants prior to sample collection. And the experiments with human samples conformed to the guidelines set by the Declaration of Helsinki.

### EMRG acquisition

All 11 human metabolism‐related pathways (Table 1) were downloaded from the Reactome database. The duplicated genes were filtered out, leaving 584 EMRGs. The relevant information of four genes was missing in the BLCA dataset from the TCGA database. Finally, 580 EMRGs were selected as candidate factors for subsequent analyses.

**Table 1:** Pathways involved in energy metabolism in the Reactome database.

Metabolic pathway	Pathway ID	Gene count
Biological oxidations	R‐HSA‐211859	213
Metabolism of carbohydrates	R‐HSA‐71387	291
Mitochondrial fatty acid beta‐oxidation	R‐HSA‐77289	37
Glycogen synthesis	R‐HSA‐3322077	16
Glycogen metabolism	R‐HSA‐8982491	27
Glucose metabolism	R‐HSA‐70326	90
Glycogen breakdown (glycogenolysis)	R‐HSA‐70221	15
Glycolysis	R‐HSA‐70171	71
Pyruvate metabolism	R‐HSA‐70268	31
Pyruvate metabolism and citric acid cycle	R‐HSA‐71406	55
Citric acid cycle	R‐HSA‐71403	22
Sum	868 (unique, 584)	

### Construction of the risk score model

Univariate Cox proportional hazard regression analysis was performed to initially identify EMRGs that were significantly involved in the OS of BLCA patients. These OS‐related EMRGs were then placed into the least absolute shrinkage and selection operator (LASSO) to remove false‐positives due to overfitting [18]. Finally, the optimal OS‐related EMRGS obtained by LASSO analysis was incorporated into the multivariate Cox proportional hazard regression analysis to construct a risk score model. Then, on the basis of a linear combination of gene expression levels and regression coefficients produced from the multivariate analysis, a risk score model for EMRGs was developed. The formula is as follows: the risk score = $\sum_{i=1}^{n}{\text{Coef}}_{i}\times X_{i}$ (Coef_*i*_ and *X*_*i*_ symbolize the regression coefficient and expression level about each EMRG, respectively). Risk scores were calculated for each BLCA patient, and patients were classified into high‐ and low‐risk groups based on median risk scores. To assess the difference in survival between the groups, Kaplan–Meier survival analysis was subsequently performed employing the r ‘survival’ package.

### Evaluation of this risk score model

Univariate and multivariate regression analyses were used to investigate whether clinical parameters and our risk score model were independent prognostic factors for BLCA patients. After combining independent prognostic factors, a nomogram that can predict the 1‐, 3‐, and 5‐year OS rates of patients was developed using the ‘rms’ package. Calibration curves were used to determine the degree of match between the predicted and actual results. The index of concordance (*C*‐index) and time‐dependent receiver operating characteristic (ROC) curves and the area under the curve (AUC) values were used to estimate the predictive accuracy of prognostic models. Additionally, decision curve analysis (DCA) was utilized to measure the net clinical benefit of standardizing prognostic models [19].

### Exploration of the immune microenvironment based on the risk score model

To research the potential mechanisms of the interaction between EMRGs and the immune microenvironment, we used estimate tools to calculate the stromal score, immune score, and estimate score. Using the cibersort algorithm, we calculated the proportion of 22 kinds of immune cells in the immune microenvironment of the two groups [20]. Spearman correlation analysis was used to confirm the connection between the risk score model and subgroups of infiltrating immune cells. Finally, we assessed the differences in checkpoint genes acquired from other studies between the groups [16, 21, 22].

### Functional analysis

The mechanism of EMRGs in the progression of BLCA was investigated using functional analysis. First, differentially expressed EMRGs between the high‐risk group and low‐risk group were screened by the ‘limma’ package and Wilcoxon test in the r software (version 4.0.4, Lucent Technologies, Mount Jasmine, NJ, USA). |Log_2_ fold‐change [FC]| > 1, false discovery rate (FDR) < 0.05 and *P* < 0.05 were the criteria for determining differentially expressed EMRGs. Then, Gene Ontology (GO) [23] functional enrichment analysis and Kyoto Encyclopedia of Genes and Genomes (KEGG) pathway analysis of differentially expressed EMRGs were carried out by the ‘clusterprofiler’ r package. In addition, gsea v4.1.0 software was run to reveal the potential biological pathways and mechanisms of the groups. Gene sets with *P* < 0.05 and FDR < 0.25 after 1000 permutations were considered to be significantly enriched [24].

### Verification of the expression of genes in the risk score model

Data from the HPA database (http://www.proteinatlas.org) [25] were chosen to verify the protein expression of the EMRGs from our risk score model in BLCA and normal control tissues. In addition, using our single‐cell RNA‐seq BLCA datasets, we also verified whether the EMRGs were expressed at the single‐cell level. Single‐cell suspensions of tissue samples were prepared via a mechanical combined enzyme digestion method, and peripheral blood mononuclear cells and granulocytes were separated through a density gradient centrifugation method. Single‐cell sequencing was performed by an Illumina NovaSeq S6000 sequencing machine (Illumina, San Diego, CA, USA) on the 10× Genomics Chromium platform, and cell ranger software (10x Genomics, Princeton, CA, USA) was used to preprocess the sequencing data. A heatmap of the expression levels of the EMRGS was drawn using the ‘complexheatmap’ and ‘pheatmap’ packages.

### Statistical analysis

All data analyses were carried out in r software (version 4.0.4). Statistical tests were two‐sided, and *P* < 0.05 was regarded as statistically significant.

## Results

### Identification of EMRGs associated with BLCA patient OS

**Fig. 1:**
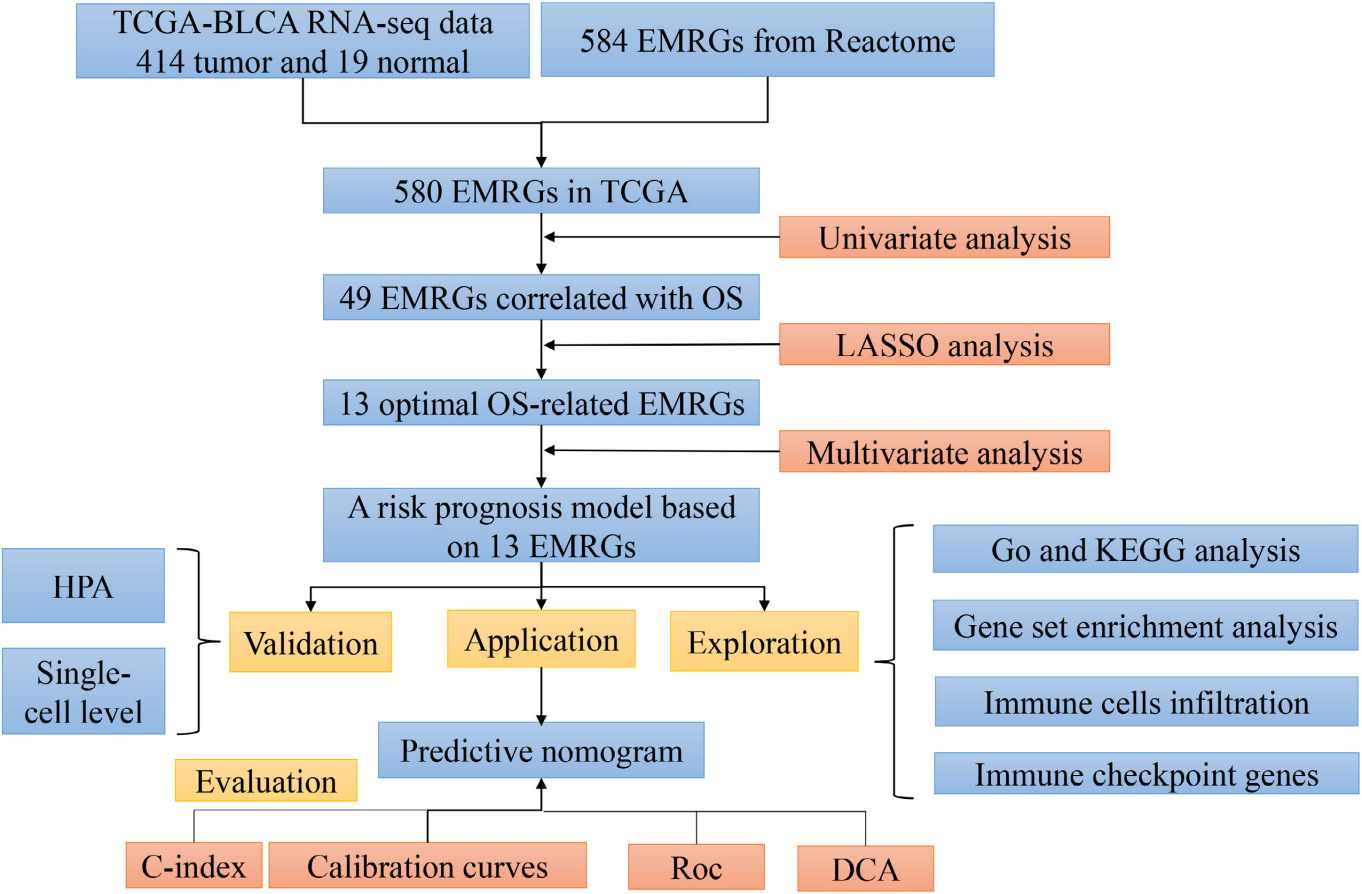
Workflow of the study design.

**Fig. 2:**
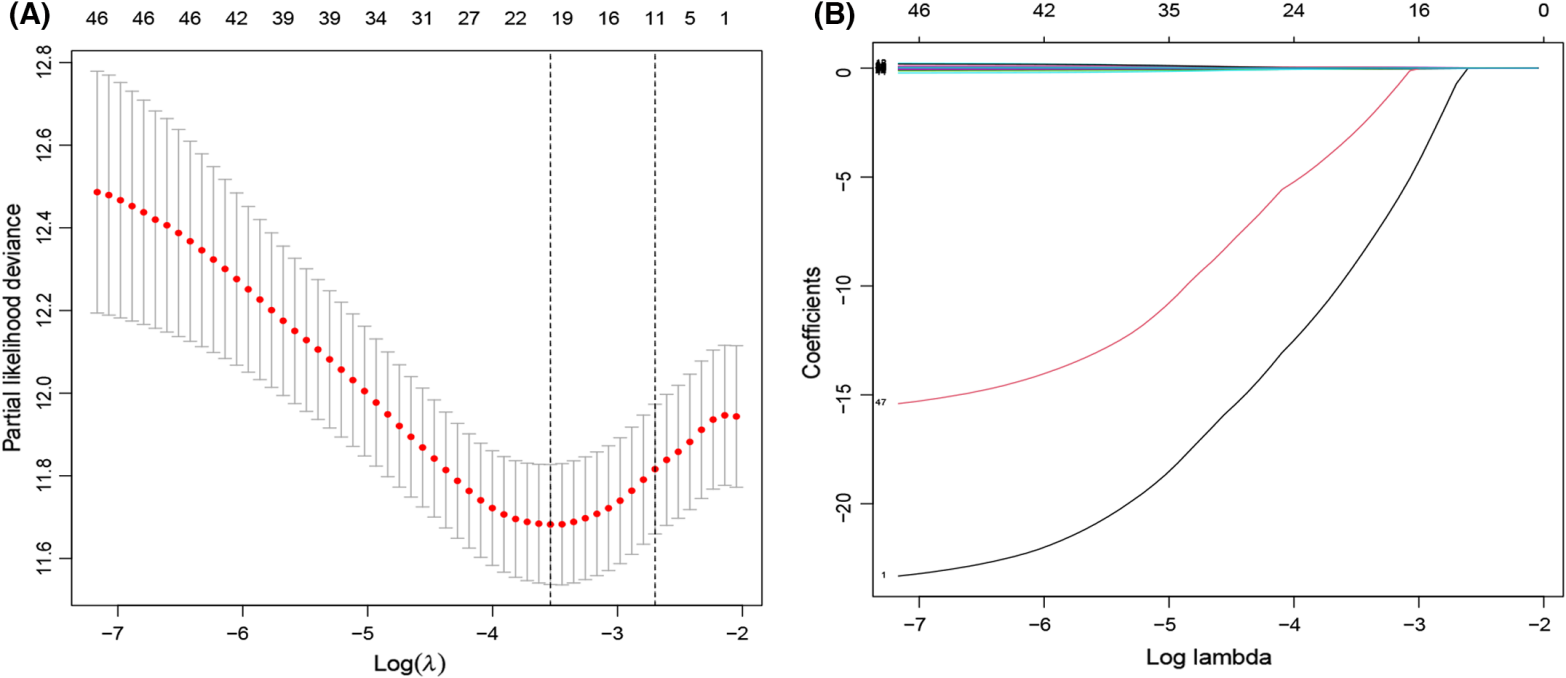
Least absolute shrinkage and selection operator regression analysis. (A) LASSO coefficient profiles of 49 EMRGs in TCGA‐BLCA data. (B) A coefficient profile plot was produced against the log(λ) sequence.

The workflow of the research is shown in Fig. 1. Univariate analysis confirmed that 49 EMRGs were associated with OS, and have been described as prognostic genes (Table S1). Subsequently, the 13 optimal OS‐related EMRGs (*ACSM2A*, *ESD*, *GALK1*, *GLCE*, *HSPG2*, *HYAL3*, *IDUA*, *MAT2B*, *MECR*, *NR1H4*, *NUP188*, *PPP2CB*, and *TPST1*) were determined using LASSO regression analysis (Fig. 2). Multivariate analysis determined that all 13 genes could be used as independent prognostic factors (Table 2).

**Table 2:** Multivariate analysis results of genes based on TCGA‐BLCA data. CI, confidence interval; HR, hazard ratio.

Genes	Coefficient	HR	95% CI of HR	*P*
*ACSM2A*	−22.353	0.000	0.000–0.002	0.007
*ESD*	0.031	1.031	1.012–1.051	0.002
*GALK1*	0.035	1.035	1.002–1.070	0.040
*GLCE*	0.061	1.063	1.011–1.118	0.017
*HSPG2*	0.015	1.016	0.996–1.035	0.119
*HYAL3*	0.056	1.058	0.990–1.130	0.095
*IDUA*	−0.049	0.952	0.904–1.002	0.061
*MAT2B*	−0.076	0.927	0.887–0.969	0.001
*MECR*	0.058	1.060	0.982–1.144	0.136
*NR1H4*	−0.094	0.910	0.824–1.005	0.062
*NUP188*	0.029	1.030	0.993–1.068	0.115
*PPP2CB*	0.027	1.027	0.995–1.060	0.099
*TPST1*	0.022	1.022	0.999–1.045	0.062

### Establishment and validation of the risk score model

**Fig. 3:**
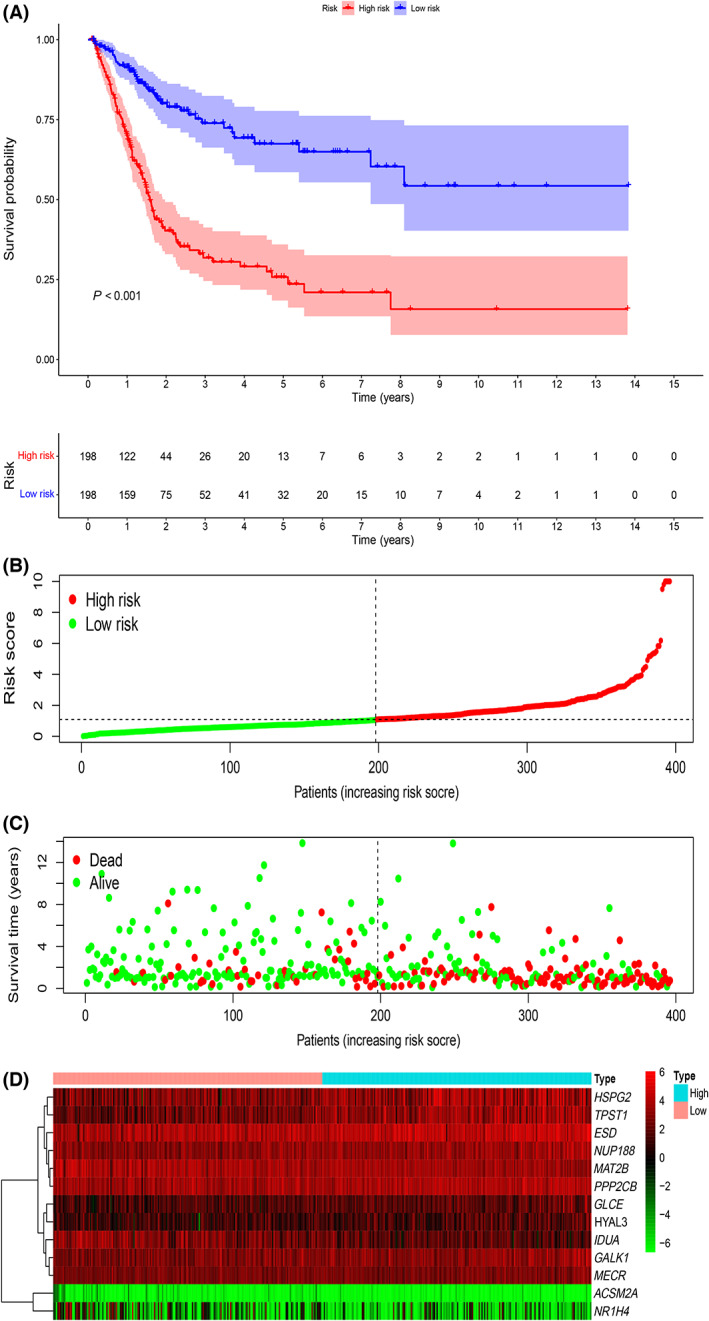
Establishment of the risk score model. (A) Kaplan–Meier curve analysis of the high‐risk and low‐risk groups. (B) The risk score distribution of patients. (C) Survival status scatterplots of patients. (D) Expression patterns of the 13 EMRGs.

**Fig. 4:**
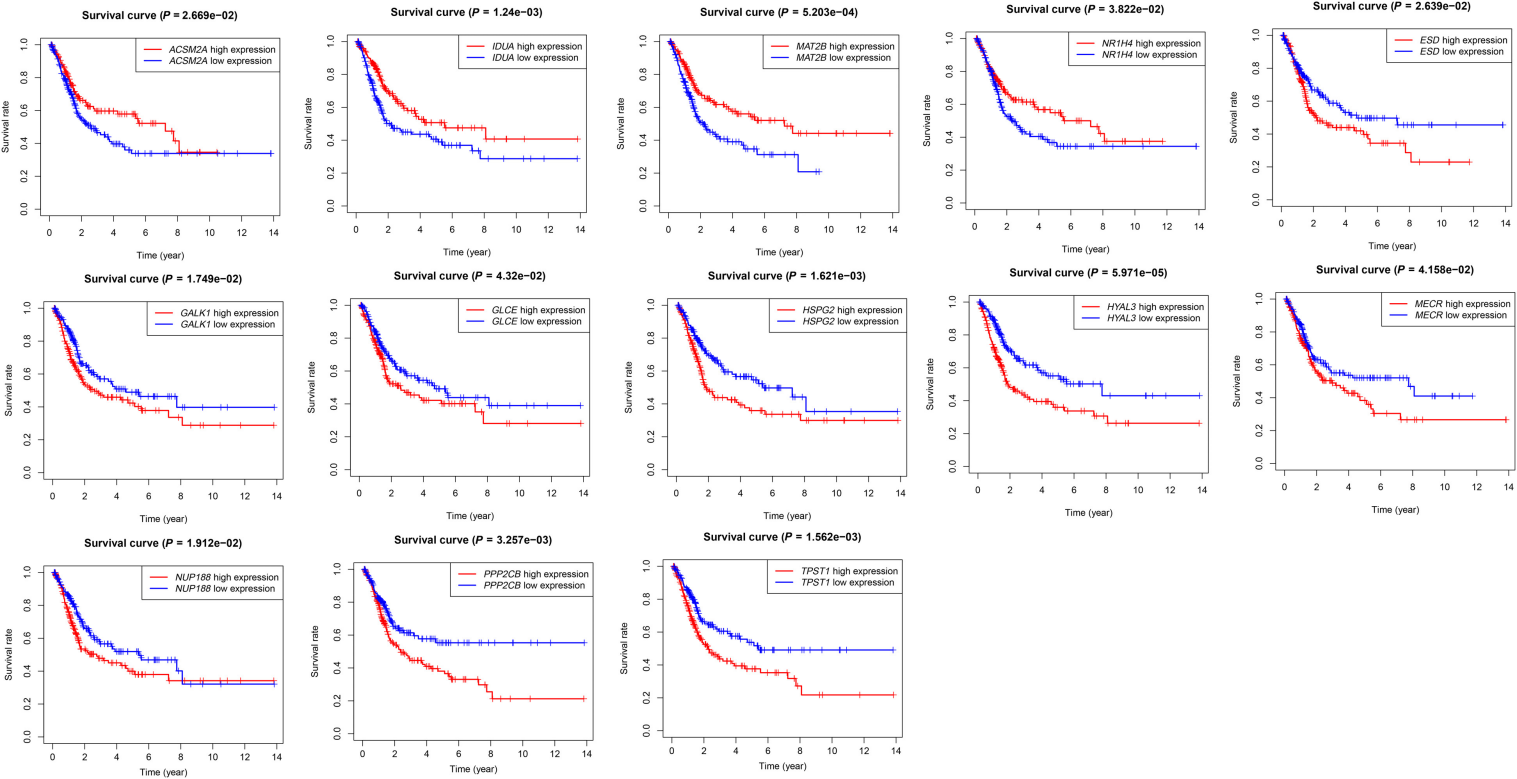
Kaplan–Meier survival analysis of the 13 EMRGs in the TCGA‐BLCA cohort.

According to the expression levels and regression coefficients of 13 EMRGs, we built a risk score model. Using the median risk score as the boundary, BLCA patients were divided into high‐risk and low‐risk groups. As shown in Fig. 3A, patients in the low‐risk group fared better than those in the high‐risk group in terms of survival rates (*P* < 0.001). We ranked the patients' risk scores from lowest to highest (Fig. 3B). The scatter plot showed that higher risk scores were associated with more deaths (Fig. 3C). We observed that the expression levels of nine genes, including *ESD*, *GALK1*, *GLCE*, *HSPG2*, *HYAL3*, *MECR*, *NUP188*, *PPP2CB*, and *TPST1*, increased with increasing risk score (Fig. 3D). The higher the expression levels of ACSM2A, IDUA, MAT2B, and NR1H4, the better the prognosis of patients with BLCA, on the contrary, the higher the expression levels of the other nine genes, the worse the prognosis of patients (all *P* < 0.05, Fig. 4).

### Evaluation of the risk score model

**Fig. 5:**
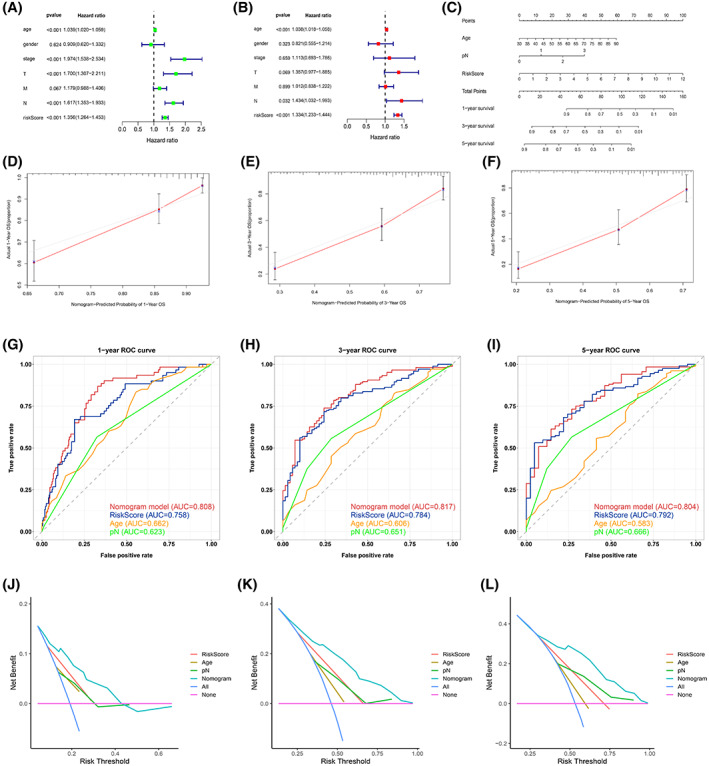
Prognostic value of the risk score model. (A) Univariate and (B) multivariate Cox proportional hazard regression analyses. (C) Nomogram integrating age, pathological N stage, and the risk score. The total points of each patient provide the estimated 1‐, 3‐, and 5‐year OS. (D–F) Calibration curve for predicting OS at 1, 3, and 5 years. The error bars indicate 95% confidence intervals. (G–I) The 1‐, 3‐, and 5‐year time‐dependent ROC curves of the nomogram, risk score, age, and pathological N stage. (J–L) The 1‐, 3‐, and 5‐year DCA curves. The *x*‐axis indicates the percentage of threshold probability, and the *y*‐axis represents the net benefit.

Univariate analysis revealed that the risk score, age, stage, and T and N stage were significantly relevant to the OS of BLCA patients (Fig. 5A). However, the results of the multiple factor analysis indicated that the risk score (hazard ratio, 1.33; 95% CI, 1.23–1.44; *P* = 6.86 × 10^−13^) was an independent and important prognostic factor (Fig. 5B). For clinical application and visualization of the risk score model, a nomogram predicting patients' 1‐, 3‐, and 5‐year OS was built by combining three prognostic factors (risk score, age, and N stage) (Fig. 5C).

The *C*‐index of the nomogram was 0.752. As shown in the calibration curves, the survival status expected by using the nomogram matched nicely with the actual outcomes (Fig. 5D–F). The AUCs of the risk score model for 1‐, 3‐, and 5‐year OS were 0.758, 0.784, and 0.792, respectively, and those of the nomogram models were 0.808, 0.817, and 0.804, respectively (Fig. 5G–I). At each survival time point, in contrast with the traditional clinical factors (age and N stage), the risk score models had the greatest prediction performance and net benefit (Fig. 5J–L).

### Relationships between the risk score model and the tumor microenvironment

**Fig. 6:**
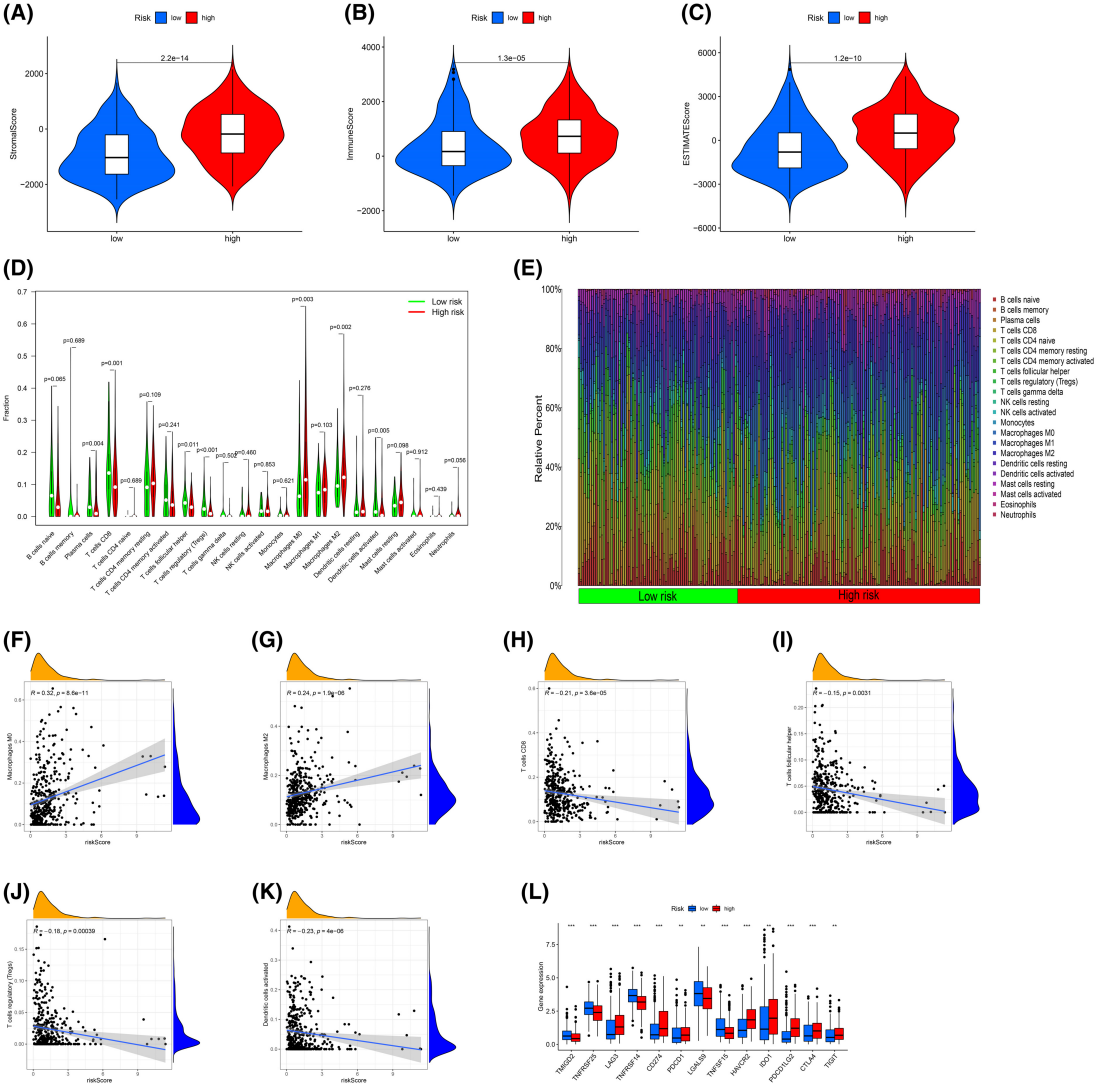
Tumor microenvironment, immune infiltration, and immune checkpoint analyses. Differences in (A) stromal score, (B) immune score, and (C) estimate score between the high‐ and low‐risk groups. Wilcoxon test. (D) Violin plot analysis comparing the distribution of 22 types of immune cell infiltrations in the groups. Wilcoxon test. (E) The ratio of 22 types of immune cells is shown for each BLCA patient by a histogram. (F–K) Spearman correlation analysis between the risk score model and immune cell infiltration levels. (L) The expression distributions of 13 immune checkpoint genes between the groups. *P < 0.05, **P < 0.01, ***P < 0.001, Wilcoxon test.

The tumor microenvironments of the entire cohort were analyzed. The stromal, immune, and estimated scores of the high‐risk group were significantly higher than those of the low‐risk group, and the differences were statistically significant (Fig. 6A–C).

The tumor microenvironment, particularly immune cells, has a significant impact on tumor patient prognosis [26, 27, 28]. We calculated the proportion of 22 immune cell types in all BLCA patients. As shown in Fig. 6D, there were significant differences in the levels of infiltration of seven different types of immune cells between the two groups. Compared with the low‐risk group, the infiltration levels of M0 macrophages (*P* = 0.003) and M2 macrophages (*P* = 0.002) in the high‐risk group were higher. Compared with the high‐risk group, the infiltration levels of plasma cells (*P* = 0.004), CD8 T cells (*P* = 0.001), follicular helper T cells (*P* = 0.011), regulatory T cells (Tregs) (*P* < 0.001), and dendritic activating cells (*P* = 0.005) were higher in the low‐risk group. In addition, the distribution of 22 immune cells was different in each BLCA patient (Fig. 6E).

Based on the consequences of Spearman correlation analysis, the infiltration levels of M0 macrophages (*R* = 0.32, *P* < 0.001, Fig. 6F) and M2 macrophages (*R* = 0.24, *P* < 0.001, Fig. 6G) were positively correlated with the risk score, whereas the infiltration levels of CD8 T cells (*R* = −0.21, *P* < 0.001, Fig. 6H), follicular helper T cells (*R* = −0.15, *P* = 0.003, Fig. 6I), Tregs (*R* = −0.18, *P* < 0.001, Fig. 6J), and dendritic activating cells (*R* = −0.23, *P* < 0.001, Fig. 6K) were negatively correlated.

Given that checkpoint inhibitors play a vital role in immunotherapy, we further explored the differences in the expression levels of immune checkpoints between different risk populations. In contrast to the low‐risk group, the expression levels of *TMIGD2*, *TNFRSF25*, *TNFRSF14*, *LGALS9*, and *TNFSF15* decreased in the high‐risk group, and those of *LAG3*, *CD274 (PD‐L1)*, *PDCD1 (PD‐1)*, *HAVCR2*, *IDO1*, *PDCD1LG2 (PD‐L2)*, *CTLA4*, and *TIGIT* were greater in the high‐risk group (Fig. 6L).

### Functional analysis

**Fig. 7:**
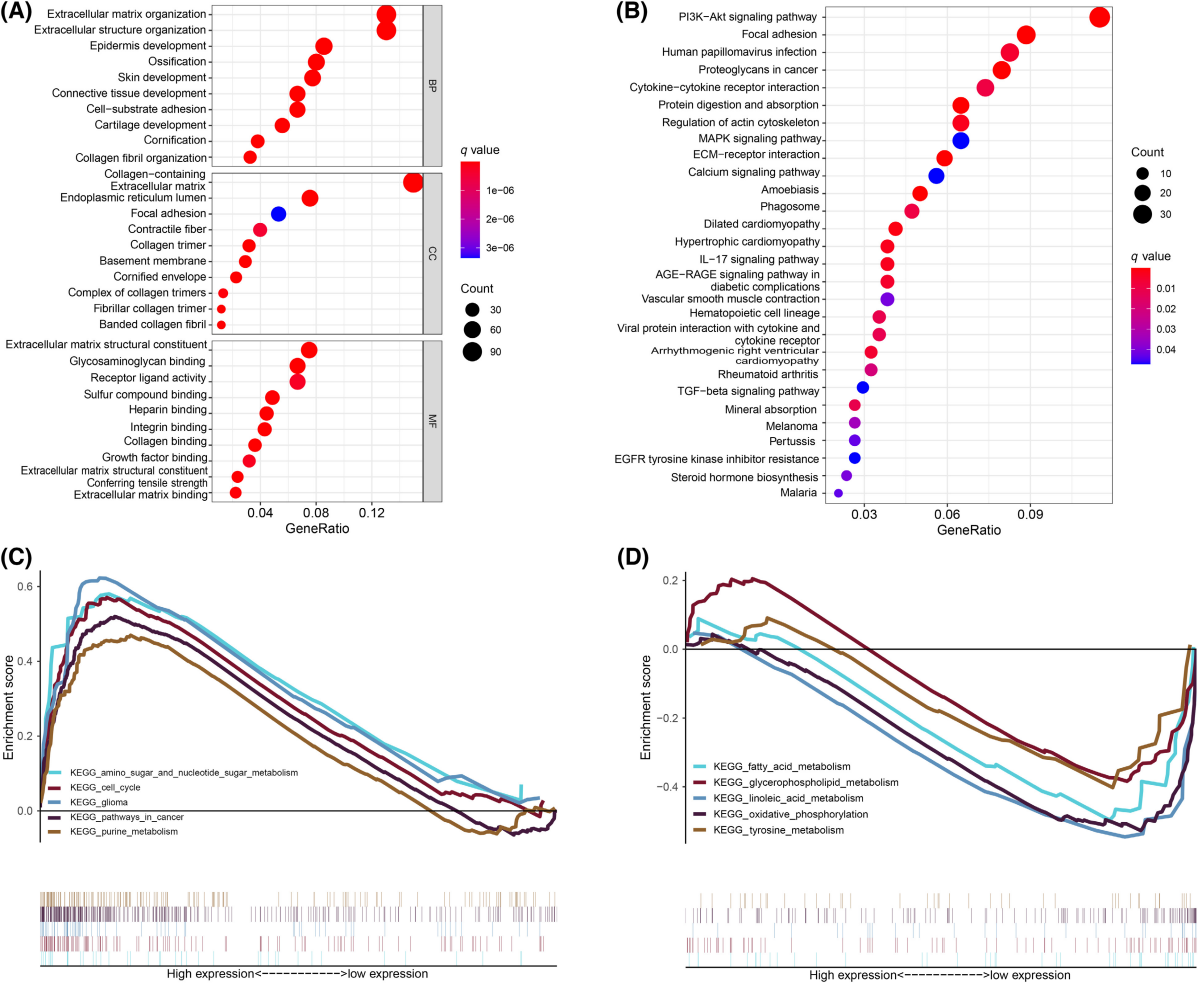
Functional analysis between the high‐risk and low‐risk groups. (A) GO analysis of DE‐EMRGs, including CC, BP, and MF. (B) KEGG analysis of DE‐EMRGs. (C) The pathways enriched in the high‐risk group. (D) The pathways enriched in the low‐risk group.

In the high‐risk and low‐risk groups, a total of 945 differentially expressed EMRGs were identified (Table S2). The GO analysis results confirmed that the differentially expressed EMRGs were significantly enriched in pathways closely associated with cell proliferation, tumor progression, and migration, such as skin development, epidermis development, and cell‐substrate adhesion (Fig. 7A). Additionally, the differentially expressed EMRGs were significantly enriched in tumor‐related pathways in KEGG pathway analysis, such as the PI3K‐Akt signaling pathway, focal adhesion and proteoglycans in cancer (Fig. 7B).

To investigate the differences in biological function among BLCA patients at different risks, we performed a gene set enrichment analysis. Multiple tumor‐related pathways were enriched in the high‐risk group, such as glioma (NES = 2.24, *P* = 0.000), pathways in cancer (NES = 2.14, *P* = 0.000), amino sugar and nucleotide sugar metabolism (NES = 1.92, *P* = 0.002), cell cycle (NES = 1.85, *P* = 0.019), and purine metabolism (NES = 1.84, *P* = 0.002) (Fig. 7C). In addition, metabolism‐related pathways, such as linoleic acid metabolism (NES = −1.74, *P* = 0.006), oxidative phosphorylation (NES = −1.69, *P* = 0.029), fatty acid metabolism (NES = −1.66, *P* = 0.029), glycerophospholipid metabolism (NES = −1.56, *P* = 0.008) and tyrosine metabolism (NES = −1.48, *P* = 0.038), were enriched in the low‐risk group (Fig. 7D).

### Verification of the expression of genes in the risk score model

**Fig. 8:**
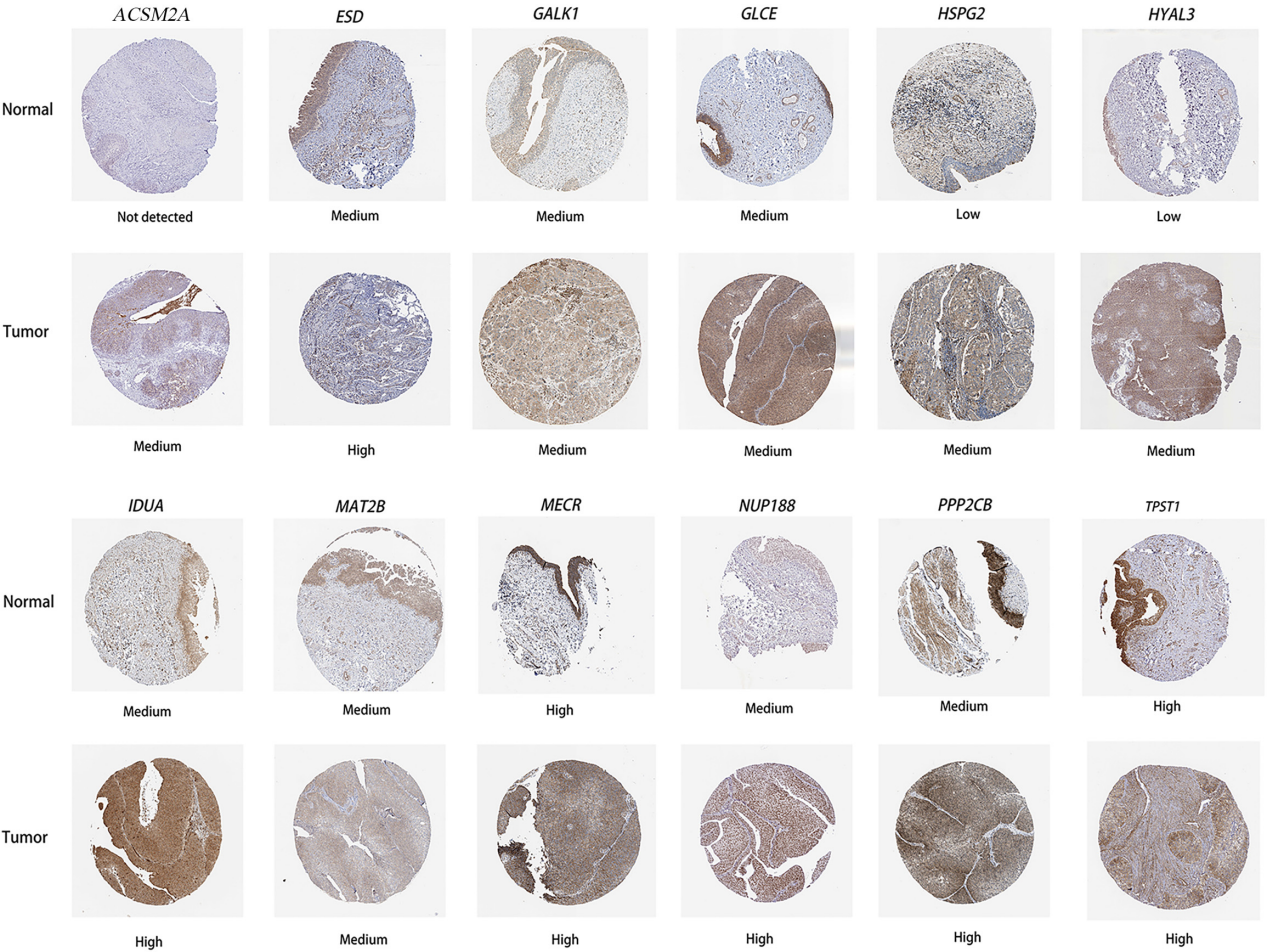
Immunohistochemistry results of 13 EMRGs in BLCA and normal tissues based on HPA.

To verify the expression levels of these 13 EMRGs (note that NR1H4 was absent), we detected the protein levels of normal samples and BLCA tissues on the HPA website (Fig. 8). In the HPA data, the protein expression levels of *ACSM2A*, *ESD*, *HSPG2*, *HYAL3*, *IDUA*, *NUP188*, and *PPP2CB in BLCA* tissues were higher than those in normal bladder tissues.

**Fig. 9:**
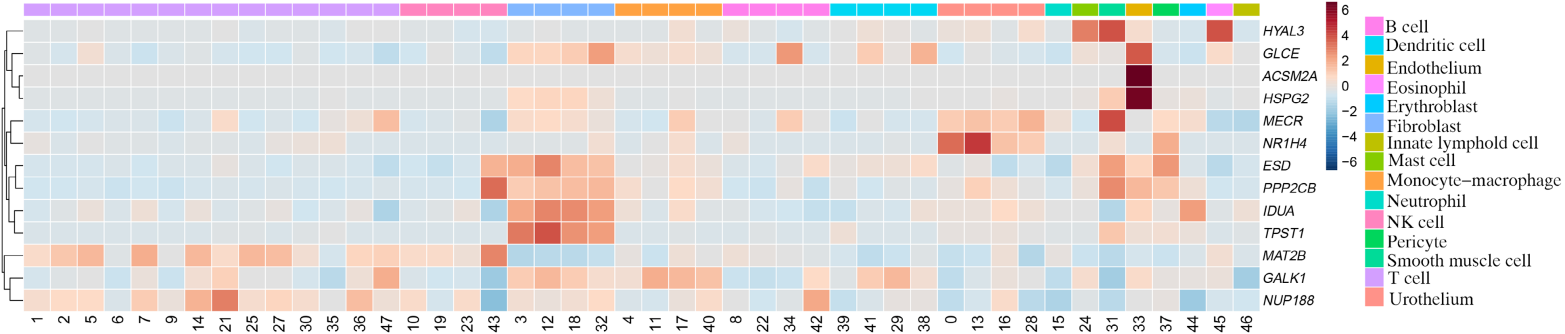
A heatmap of the 13 EMRGs at the bladder cancer single‐cell level.

To further validate the expression levels of the 13 EMRGs in single cells, our BLCA scRNA‐seq dataset was analyzed (Fig. 9). The results showed that GALK1 and NUP188 were expressed in most cell types, such as T cells, fibroblasts, monocyte‐macrophages, and B cells. *TPST1* and *IDUA* were mainly expressed in fibroblasts. *ACSM2A* and *HSPG2* were highly expressed in the endothelium. Genes such as *HYAL3* and *MAT2B* were expressed at a low level in particular cell types.

## Discussion

Bladder urothelial carcinoma is a kind of molecular heterogeneous malignant tumor, and its molecular characteristics are closely related to the prognosis of BLCA [29]. In recent years, disorders of energy metabolism in most cancers have steadily become a research hotspot. Reprogramming of energy metabolism is one of the significant markers of cancer, promoting the rapid growth and proliferation of tumor cells [8]. Abnormal energy metabolism is closely related to the prognosis of cancer patients. Relevant studies have demonstrated that aberrant regulation of metabolic pathways such as glycolysis, oxidative metabolism, and pyruvate metabolism can affect the treatment and prognosis of BLCA patients [14, 30, 31]. As prognostic evaluation indicators, EMRGs have been studied and made some progress in malignant tumors such as esophageal cancer [32], ovarian cancer [33], and glioma [34]. However, there are few reports on EMRGs predicting prognosis in BLCA. Therefore, we designed this study to screen for potential prognostic biomarkers associated with EMRGs and explore the possible mechanisms of EMRG progression in BLCA.

Through univariate and multivariate Cox regression analysis, EMRGs related to BLCA prognosis were screened and the model was optimized. Finally, a risk score model was developed based on 13 EMRGs. Survival analysis showed that patients in the high‐risk group had shorter OS and worse prognosis. TNM staging is helpful to predict the prognosis of cancer patients. Multivariate Cox regression analysis showed that the risk score model, age, and N stage were independent prognostic factors for BLCA patients. ROC curve analysis showed that the AUC value of the risk score model was higher than that of the traditional clinical parameters (age, N stage), suggesting that the predictive efficacy of the risk score model was better than that of the commonly used clinical prognostic indicators.

Previous researchers have constructed a prediction model for BLCA prognosis based on the TCGA‐BLCA dataset. For example, Peng *et al*. [35] developed a prognostic model based on five autophagy‐related genes, and its AUC value was 0.724. Zhang *et al*. [36] constructed a prognostic model based on five pyroptosis‐related genes, and the AUCs of the 5‐gene model for 1‐, 3‐, and 5‐year OS were 0.667, 0.632, and 0.637, respectively. The AUCs of the risk score model we established to predict the 1 ‐, 3 ‐, and 5‐year OS were 0.758, 0.784, and 0.792, respectively. It is reported that AUC > 0.75 is considered to have excellent predictive value [37]. In summary, we can draw a conclusion that the risk score model related to EMRGs is superior to some previous biomarkers in OS prediction of BLCA patients, and it has great potential clinical application value.

Combined with age, N stages, and risk score, a nomogram prediction model was established. The nomogram prediction model has a good clinical application value, and its prediction efficiency (AUC = 0.817) is better than that of a single predictor. Our results also showed that the immune microenvironment differed notably among different risk groups, and eight checkpoint genes (*LAG3*, *PD‐L1*, *PD‐1*, *HAVCR2*, *IDO1*, *PD‐L2*, *CTLA4*, and *TIGIT*) were significantly upregulated in the high‐risk groups. Moreover, 13 EMRGs were mainly enriched in tumor‐related pathways and metabolism‐related pathways. Finally, the expression levels of 13 EMRGs were proven in the HPA database and our single‐cell sequencing dataset.

According to the risk score model we built, among BLCA patients, those with high expression levels of *ESD*, *GALK1*, *GLCE*, *HSPG2*, *HYAL3*, *MECR*, *NUP188*, *PPP2CB*, and *TPST1* had a poorer prognosis than those with low expression. Subsequently, our results confirmed that these genes were associated with the OS of BLCA patients. *ESD*, *GALK1*, *GLCE*, *HSPG2*, *HYAL3*, *MECR*, *NUP188*, *PPP2CB*, and *TPST1* were prognostic risk factors in patients with BLCA, whereas the others were protective factors.

The majority of the genes in our risk score model have previously been linked to cancer. However, the role of several genes in BLCA remains unknown. *GALK1* encodes galactokinase (*GALK*), and mutations in *GALK1* can lead to GALK deficiency or type 2 galactosemia [38]. In addition, *GALK*‐deficient individuals are unable to phosphorylate galactose and thus accumulate galactose and galactitol [38]. Because of its importance in protein glycosylation, researchers believe that GALK1 could be used as a new valuable therapeutic target for hepatocellular carcinoma [39]. In addition, no studies have found a relationship between *GALK1* and BLCA, but our results provide new evidence that it may serve as a meaningful marker and immunotherapeutic target for BLCA patients. *PPP2CB* was confirmed to be a risk factor for the activity, proliferation, and migration of BLCA cells [40], which corresponds to our findings. Previous studies suggested that *TPST1* upregulated expression levels in BLCA, breast cancer, tongue squamous cell carcinoma, and nasopharyngeal carcinoma [41, 42, 43, 44] and has a significant impact on the invasion and metastasis of hypopharyngeal cancer and colorectal cancer [45, 46]. Chen *et al*. [41] confirmed found that high *TPST1* expression levels in BLCA were closely linked with a low survival rate and high tumor pathological stage; therefore, *TPST1* could be used as a prognostic indicator. It has been reported that *IDUA* is involved in chondroitin sulfate/dermatan sulfate metabolism and glycosaminoglycan metabolism, and *IDUA_GNPDA1* could be used as a beneficial prognostic factor in hepatocellular carcinoma [47]. The expression level of *IDUA* in breast cancer patients was decreased compared with that in normal controls, and *IDUA* was confirmed to be a potential target for visceral metastasis in breast cancer patients [48].

The tumor microenvironment and immune cells affect the treatment and prognosis of BLCA patients. Immune cell infiltration in tumors has been shown to correlate with patient prognosis [49, 50, 51]. In the immunoassay, the high‐risk patients had higher levels of M0 and M2 macrophage infiltration than the low‐risk patients. The risk score has a positive relationship with the M0 and M2 macrophage infiltration levels. It is well known that macrophages have immunosuppressive and tumor‐promoting effects. As a result, immune function in high‐risk BLCA patients could be suppressed, which needs further validation. In our results, eight checkpoint genes were more highly expressed in the high‐risk group, and five checkpoint genes were more highly expressed. These results suggest that, depending on the characteristics of immune infiltration and checkpoint genes in BLCA patients, specific patients should be selected for inhibitor therapy so that the treatment effect will be better.

Notably, we observed that tumor‐related pathways were significantly enriched in high‐risk patients, while metabolism‐related pathways, such as fatty acid metabolism, glycerophospholipid metabolism, linoleic acid metabolism, and tyrosine metabolism pathways, were significantly enriched in low‐risk patients. Our results provide some new perspectives for the personalized treatment of BLCA. For example, in the future, low‐risk patients might benefit more from the targeted treatment of fatty acid metabolism.

Our research is the first to validate the expression levels of EMRGs of the risk score model using single‐cell sequencing data of BLCA, which is innovative, but there are also some deficiencies that need to be improved. First, because the research subjects came from Europe, we need further validation in a large global multicenter cohort. Second, other traditional clinical parameters, such as smoking history, family history, and treatment method, were not included in the analysis. At present, we cannot determine whether these factors affect the predictive effect of the risk score model. Finally, genes have a variety of biologically and pathologically relevant functions in most cancer cells and are closely related to the internal features of cancers, thus serving as precise predictors of cancer development and prognosis [52]. Therefore, it is necessary to conduct functional experimental research on 13 EMRGs of the risk score model to explore the mechanism of these genes in the occurrence, development, and prognosis of BLCA.

## Conclusion

In summary, we developed a risk score model based on 13 EMRGs with better predictive efficacy than traditional clinical parameters. The immune microenvironment differed significantly among different risk groups, and eight checkpoint genes (*LAG3*, *PD‐L1*, *PD‐1*, *HAVCR2*, *IDO1*, *PD‐L2*, *CTLA4*, and *TIGIT*) were significantly upregulated in the high‐risk groups. This could facilitate the precise selection of patients who would benefit more from immunotherapy.

## Author contributions

CH, YL, QL, CW, BF, XM, and RY collected the data, and performed data cleaning and statistical analysis. CH was accountable for writing the manuscript and preparing diagrams. YL, QL, and LZ revised the article. SH, NL, JC, and FW were accountable for the research content framework building. LL, ZM, and LM guided research design and promoted project progress. All authors approved the submitted manuscript.

## Conflict of interest

The authors declare no conflict of interest.

## Supporting information


**Table S1.** EMRGs associated with BLCA patients' OS in TCGA‐BLCA data.

**Table S2.** Differential metabolism‐related genes between the high‐ and low‐risk groups in TCGA‐BLCA data.

## Data Availability

The transcriptome data and clinical information of BLCA patients are available in TCGA (https://portal.gdc.cancer.gov). The code used in the data analysis process can be obtained from the corresponding author upon reasonable request.
