# Supplementary material for: Establishment of a risk score model for bladder urothelial carcinoma based on energy metabolism‐related genes and their relationships with immune infiltration
Source: FEBS Open Bio. 2023 Mar 5;13(4):736–50. doi: 10.1002/2211-5463.13580 (PMC10068335; doi:10.1002/2211-5463.13580)
Supplement: Supplementary file 1 — Table S1. EMRGs associated with BLCA patients' OS in TCGA‐BLCA data. [file FEB4-13-736-s002.pdf]

Supplementary Table 1 EMRGs associated with BLCA Patients' OS in TCGA-BLCA data.

Genes	B	SE	HR	95%CI of HR		*P*
				Lower limit	Upper limit	
ACSM2A	-15.785	7.757	0.000	0.000	0.560	0.042
ACSM6	-0.091	0.045	0.913	0.836	0.997	0.042
ACY1	-0.133	0.058	0.876	0.781	0.982	0.023
AOC2	-0.114	0.047	0.892	0.813	0.978	0.015
ARSB	0.170	0.066	1.186	1.041	1.351	0.010
B4GAT1	0.049	0.017	1.050	1.016	1.085	0.003
BGN	0.001	0.000	1.001	1.000	1.001	0.007
CALM1	0.013	0.005	1.013	1.004	1.023	0.006
CHPF	0.006	0.002	1.006	1.002	1.010	0.003
CHST15	0.041	0.016	1.042	1.009	1.075	0.011
CSPG4	0.016	0.006	1.016	1.004	1.029	0.011
CYP2D6	-0.520	0.236	0.594	0.374	0.943	0.027
CYP4B1	-0.003	0.001	0.997	0.994	0.999	0.012
CYP4F12	-0.031	0.012	0.969	0.948	0.992	0.008
CYP51A1	0.157	0.073	1.170	1.013	1.350	0.032
DCN	0.005	0.002	1.005	1.001	1.009	0.011
DECR1	-0.018	0.007	0.982	0.969	0.996	0.011
DSEL	0.226	0.092	1.254	1.047	1.502	0.014
EPHX1	0.002	0.001	1.002	1.000	1.004	0.042
ESD	0.031	0.009	1.032	1.014	1.049	0.000
FBP1	-0.006	0.002	0.994	0.991	0.998	0.001
FMOD	0.013	0.006	1.013	1.002	1.025	0.017
FUT11	0.076	0.038	1.079	1.001	1.164	0.046
GALK1	0.055	0.014	1.056	1.028	1.085	0.000
GLCE	0.096	0.024	1.101	1.049	1.154	0.000
GNPDA1	-0.026	0.010	0.975	0.955	0.995	0.014
HK1	0.013	0.005	1.013	1.002	1.024	0.021
HSPG2	0.027	0.008	1.027	1.011	1.044	0.001
HYAL3	0.090	0.031	1.094	1.029	1.164	0.004
IDUA	-0.084	0.023	0.920	0.879	0.963	0.000
MAOA	-0.009	0.003	0.991	0.985	0.998	0.013
MAT2B	-0.053	0.017	0.949	0.918	0.981	0.002
MECR	0.079	0.035	1.083	1.011	1.160	0.024
NR1H4	-0.106	0.054	0.900	0.810	1.000	0.049
NUP188	0.042	0.016	1.043	1.011	1.076	0.008
OGDH	0.016	0.007	1.016	1.003	1.030	0.018
OGN	0.039	0.017	1.040	1.006	1.076	0.021
PAPSS2	0.027	0.012	1.028	1.004	1.053	0.024
PFKM	0.052	0.023	1.053	1.007	1.102	0.024
PGAM1	0.012	0.006	1.012	1.001	1.024	0.032
PPP2CB	0.040	0.014	1.041	1.013	1.070	0.004
PTGIS	0.010	0.005	1.010	1.000	1.019	0.048
PYGB	0.004	0.002	1.004	1.000	1.008	0.032
SLC16A8	-0.289	0.129	0.749	0.581	0.965	0.026
SLC35B3	-0.043	0.018	0.958	0.924	0.993	0.020
ST3GAL6	0.314	0.110	1.369	1.105	1.697	0.004
SULT6B1	-17.540	8.227	0.000	0.000	0.243	0.033
TPST1	0.038	0.009	1.039	1.021	1.058	0.000
VCAN	0.011	0.005	1.011	1.002	1.021	0.015

B:regression coefficient,SE:standard error,HR:hazard ratio,CI:confidence interval.
