# Supplementary material for: Establishment of a risk score model for bladder urothelial carcinoma based on energy metabolism‐related genes and their relationships with immune infiltration
Source: FEBS Open Bio. 2023 Mar 5;13(4):736–50. doi: 10.1002/2211-5463.13580 (PMC10068335; doi:10.1002/2211-5463.13580)
Supplement: Supplementary file 2 — Table S2. Differential metabolism‐related genes between the high‐ and low‐risk groups in TCGA‐BLCA data. [file FEB4-13-736-s001.pdf]

Supplementary Table 2 Differential metabolism-related genes between the high- and low-risk groups in TCGA-BLCA data.

Genes	Mean risk score of the low-risk group	Mean risk score of the high-risk group	log2FC	*P*	FDR
SRCIN1	2.659	1.182	-1.169	6.93E-18	3.95E-16
ST6GALNAC5	0.651	1.394	1.098	3.19E-09	1.98E-08
RASL12	2.182	4.459	1.031	5.47E-07	2.13E-06
GZMA	8.22	17.955	1.127	0.00204684	0.003878383
PTGS1	3.208	10.658	1.732	1.44E-17	7.26E-16
CYP4B1	84.422	29.031	-1.54	7.49E-14	1.30E-12
PCOLCE2	0.655	2.127	1.698	1.87E-13	2.97E-12
ITGA11	1.638	4.505	1.459	6.96E-14	1.21E-12
ADCY7	0.611	1.48	1.277	2.70E-16	9.72E-15
AC004080.1	5.277	1.943	-1.441	4.90E-10	3.62E-09
TRIM31	21.074	5.141	-2.035	3.40E-12	4.00E-11
IGHV3-7	1.255	2.64	1.073	0.000293157	0.000662981
MSRB3	2.378	6.549	1.461	4.39E-15	1.07E-13
COL6A3	15.192	41.936	1.465	1.30E-18	9.50E-17
COLEC12	1.039	2.877	1.469	3.79E-13	5.62E-12
VSTM2L	1.802	7.046	1.967	1.39E-05	4.12E-05
CDK6	1.844	4.139	1.167	7.14E-15	1.67E-13
ITGA7	2.268	4.754	1.068	0.000104559	0.000259077
PSD	1.01	2.432	1.268	0.000160964	0.000383916
BHLHE41	26.787	13.37	-1.003	5.88E-15	1.38E-13
TMC7	5.327	2.319	-1.2	4.65E-18	2.85E-16
FOXD1	0.98	2.149	1.133	1.36E-09	9.20E-09
IGFL2	0.911	4.79	2.395	2.68E-08	1.35E-07
RPL21P13	2.011	0.984	-1.031	0.005220337	0.009085468
SGK2	3.937	1.552	-1.343	7.34E-14	1.27E-12
AL731567.1	4.237	1.7	-1.318	2.04E-18	1.46E-16
VIM	61.326	140.539	1.196	1.35E-14	2.83E-13
MIR4768	5.827	2.707	-1.106	0.001113442	0.002225516

MT-ND1	6003.663	2873.898	-1.063	2.12E-14	4.20E-13
MYADM	15.914	33.314	1.066	1.54E-14	3.17E-13
UPK1A	198.673	98.588	-1.011	3.40E-09	2.09E-08
CPXM2	2.235	4.86	1.121	1.64E-07	7.13E-07
FBN1	2.289	6.55	1.517	2.78E-20	3.61E-18
GSDMB	13.345	5.351	-1.318	2.42E-22	8.28E-20
AATBC	8.016	2.707	-1.566	6.59E-20	7.45E-18
RBP1	3.969	14.656	1.885	5.30E-13	7.59E-12
PLIN5	4.047	1.057	-1.937	2.55E-23	1.66E-20
AC104695.2	3.188	1.408	-1.179	2.31E-13	3.58E-12
ZNF66	1.908	0.873	-1.128	1.37E-11	1.40E-10
SSC5D	1.233	2.82	1.193	4.81E-13	6.95E-12
LMOD1	5.86	18.027	1.621	1.14E-05	3.44E-05
COL16A1	5.357	13.102	1.29	7.27E-19	5.76E-17
PVALB	22.222	2.824	-2.976	6.35E-05	0.000164625
HSPB6	5.077	18.814	1.89	1.06E-06	3.87E-06
SIDT1	2.493	1.146	-1.122	9.27E-14	1.57E-12
EPHB6	27.878	11.086	-1.33	1.30E-12	1.70E-11
MTND2P28	341.535	155.742	-1.133	1.28E-12	1.69E-11
FOXA1	30.023	13.301	-1.175	1.39E-20	2.13E-18
AL035446.1	0.706	1.526	1.112	4.31E-08	2.10E-07
COL8A2	2.547	6.154	1.273	3.44E-16	1.19E-14
SNCG	414.347	181.32	-1.192	1.33E-15	3.82E-14
BCAS1	15.292	5.472	-1.483	3.31E-15	8.45E-14
KRT4	24.572	54.092	1.138	0.02855374	0.042127898
NT5E	4.697	12.318	1.391	5.41E-10	3.96E-09
ATF7IP2	5.564	2.414	-1.205	6.61E-19	5.31E-17
ADAMTS2	3.487	8.491	1.284	5.55E-18	3.31E-16
GJB2	63.338	170.045	1.425	1.64E-06	5.77E-06
AC023421.2	1.756	0.451	-1.963	2.06E-18	1.46E-16
B4GALT6	1.968	0.947	-1.055	0.001872823	0.00357784
LINC01481	1.603	0.775	-1.049	3.24E-13	4.85E-12

THBS4	0.779	2.002	1.363	7.97E-05	0.000202917
CXCL12	2.878	8.311	1.53	2.73E-12	3.29E-11
DUSP2	30.019	10.107	-1.57	2.86E-11	2.72E-10
ENGASE	14.681	6.735	-1.124	5.15E-23	2.84E-20
KCCAT333	20.157	6.871	-1.553	3.73E-15	9.25E-14
HSPB7	1.33	4.373	1.717	5.07E-09	3.00E-08
AC003070.1	4.76	2.144	-1.151	5.92E-19	4.81E-17
ADGRA2	2.888	5.809	1.008	1.37E-09	9.27E-09
KRT7-AS	16.07	6.622	-1.279	2.18E-17	1.06E-15
AC104825.1	3.309	1.52	-1.122	6.53E-17	2.79E-15
MIR3677	1.469	0.704	-1.061	2.17E-08	1.12E-07
GPR78	1.801	0.847	-1.088	0.001392564	0.002729308
AL355353.1	8.24	3.342	-1.302	2.41E-17	1.15E-15
MT-ATP8	3943.682	1938.562	-1.025	3.86E-13	5.71E-12
THBS1	21.809	52.418	1.265	2.07E-15	5.63E-14
ACTG2	27.184	101.899	1.906	1.28E-08	6.90E-08
AZGP1	5.813	1.632	-1.833	6.93E-12	7.51E-11
RGL3	9.36	3.983	-1.233	2.19E-12	2.72E-11
SPRR2F	1.192	3.376	1.502	0.000115647	0.00028374
S100A10	152.045	349.136	1.199	5.58E-21	1.09E-18
LAMC2	23.393	82.304	1.815	2.26E-06	7.75E-06
PI16	1.399	3.392	1.278	0.006093865	0.010447773
DPYSL3	9.625	26.403	1.456	5.11E-15	1.22E-13
SNAI2	11.773	25.884	1.137	1.65E-10	1.34E-09
ATP6V0CP2	1.694	0.591	-1.519	2.74E-12	3.29E-11
CDK5R1	1.315	2.983	1.182	1.30E-06	4.69E-06
ATOH8	3.747	0.947	-1.984	1.34E-14	2.81E-13
PID1	0.668	1.559	1.222	5.91E-09	3.43E-08
CSF1R	5.174	11.564	1.16	1.02E-12	1.36E-11
CHI3L1	14.61	45.84	1.65	4.73E-12	5.34E-11
ASPN	4.78	12.535	1.391	3.88E-12	4.46E-11
RNF186	1.699	0.5	-1.764	6.43E-09	3.70E-08

MT-RNR1	1828.308	833.864	-1.133	2.81E-18	1.84E-16
AC010735.2	3.027	1.411	-1.101	1.44E-05	4.25E-05
JAM3	2.196	4.817	1.133	1.53E-08	8.14E-08
MT-TL1	9.416	2.93	-1.684	4.28E-12	4.88E-11
GXYLT2	0.647	1.993	1.623	5.24E-23	2.84E-20
HMGA2	0.435	2.29	2.395	2.06E-12	2.58E-11
FLNC	2.242	10.188	2.184	6.48E-17	2.79E-15
INA	19.291	9.622	-1.004	6.56E-07	2.51E-06
CASC22	2.098	0.149	-3.819	1.60E-20	2.40E-18
GPR34	1.129	2.27	1.008	1.09E-10	9.18E-10
MUC4	1.906	3.867	1.021	0.004497199	0.007920185
SERPINB7	0.654	4.2	2.683	2.82E-13	4.29E-12
ACTC1	2.91	12.033	2.048	3.56E-09	2.18E-08
UGT2B7	3.984	1.984	-1.006	2.73E-07	1.13E-06
SNX31	35.31	13.532	-1.384	9.68E-16	2.89E-14
CPXM1	7.275	21.892	1.589	1.91E-14	3.85E-13
FOLR2	5.467	13.737	1.329	1.65E-09	1.09E-08
SPRR2E	5.076	33.672	2.73	1.31E-08	7.04E-08
EREG	2.113	7.531	1.834	8.68E-07	3.24E-06
ERVE-1	3.101	1.333	-1.218	5.48E-13	7.79E-12
PLXNB3	4.438	2.189	-1.019	4.88E-06	1.58E-05
IL20RB	6.037	14.094	1.223	0.002004959	0.003806233
FAHD2P1	2.86	0.731	-1.969	1.22E-13	2.03E-12
NOD2	0.611	1.543	1.336	1.14E-12	1.51E-11
BX470102.1	14.136	5.116	-1.466	4.70E-15	1.13E-13
CDH2	0.641	2.265	1.821	1.24E-11	1.27E-10
SHH	7.754	2.06	-1.912	7.24E-12	7.81E-11
MAP3K20	2.289	5.044	1.14	1.05E-23	9.11E-21
ARSI	1.161	4.279	1.883	3.35E-25	5.44E-22
KRT20	139.956	59.038	-1.245	9.24E-08	4.21E-07
PALLD	8.901	20.434	1.199	1.83E-22	6.82E-20
LINC01612	3.428	1.313	-1.385	9.65E-12	1.01E-10

TBX3	44.825	17.03	-1.396	8.33E-21	1.44E-18
SCG2	0.458	1.997	2.125	8.70E-13	1.18E-11
SLC39A14	4.055	8.489	1.066	8.33E-21	1.44E-18
CST6	25.577	64.073	1.325	0.000415106	0.000908442
HOXB3	6.548	2.642	-1.309	3.22E-16	1.13E-14
LRMP	7.223	3.494	-1.048	4.60E-11	4.17E-10
NOTUM	0.682	23.38	5.099	0.023756981	0.035716564
HAVCR1	2.992	1.128	-1.408	1.63E-07	7.11E-07
CLIC6	10.328	3.694	-1.483	0.001828771	0.003501913
NKD1	0.31	2.601	3.07	2.71E-05	7.54E-05
PLA2G6	3.625	1.736	-1.062	4.57E-21	9.14E-19
ANXA6	10.637	23.468	1.142	1.07E-17	5.71E-16
HYAL1	0.677	1.772	1.389	6.91E-08	3.22E-07
C8orf88	0.902	2.202	1.287	1.81E-12	2.29E-11
KLK10	1.511	12.348	3.031	3.58E-09	2.19E-08
FSTL3	10.977	25.029	1.189	2.57E-12	3.12E-11
TWIST1	2.428	5.949	1.293	1.66E-15	4.66E-14
KRT75	1.09	3.025	1.473	4.03E-08	1.98E-07
ANPEP	2.744	14.828	2.434	1.61E-12	2.07E-11
CNN1	15.397	56.46	1.875	7.63E-09	4.32E-08
PCDHGC3	1.401	2.883	1.041	2.24E-12	2.76E-11
GATA6	1.011	2.166	1.099	3.00E-11	2.84E-10
AP001207.3	10.886	5.139	-1.083	5.04E-07	1.98E-06
SLC6A14	1.357	3.119	1.201	0.021635598	0.032815462
RAMP1	7.333	18.131	1.306	4.53E-12	5.13E-11
TPSP2	5.521	2.75	-1.006	6.41E-07	2.46E-06
EFHD1	1.87	5.014	1.423	0.001550771	0.003012147
SERPINE1	58.765	123.297	1.069	2.60E-10	2.05E-09
CERCAM	10.331	22.415	1.117	4.88E-20	5.77E-18
VMO1	2.716	5.847	1.106	1.45E-11	1.47E-10
AC105460.1	3.776	8.011	1.085	0.004567	0.008028981
RBBP8NL	8.444	4.13	-1.032	3.64E-15	9.06E-14

UGT2B15	4.801	0.823	-2.544	4.60E-16	1.53E-14
CTSV	2.362	7.147	1.597	5.00E-13	7.17E-12
VSIG10L	1.843	4.218	1.194	0.007336016	0.012341511
FAHD2CP	4.726	1.854	-1.35	2.13E-13	3.34E-12
AC018904.1	11.736	5.345	-1.135	2.73E-18	1.79E-16
SERPINB2	3.961	12.325	1.638	6.15E-10	4.46E-09
DIXDC1	0.825	1.841	1.158	1.20E-15	3.49E-14
PPP1R14C	4.474	10.471	1.227	3.95E-07	1.59E-06
CCL20	7.464	16.676	1.16	0.006360206	0.010858602
SBSN	5.001	39.664	2.988	2.46E-06	8.39E-06
ITLN1	46.638	4.94	-3.239	0.000723988	0.001509973
CSF3	2.076	4.667	1.169	6.72E-07	2.57E-06
PAQR8	6.307	2.889	-1.126	1.76E-08	9.24E-08
GATA3-AS1	9.379	2.82	-1.734	5.38E-20	6.19E-18
MT-TF	2.992	1.421	-1.074	1.77E-05	5.12E-05
SYT11	1.74	3.612	1.054	4.34E-11	3.95E-10
GAS1	2.56	7.72	1.592	4.18E-19	3.60E-17
PPFIBP2	12.366	4.713	-1.392	7.70E-23	3.51E-20
IGFBP5	29.708	60.658	1.03	3.26E-08	1.62E-07
TFF1	47.276	12.64	-1.903	4.57E-08	2.21E-07
COL6A2	66.01	188.062	1.51	1.83E-21	4.25E-19
COL1A1	216.556	622.364	1.523	1.04E-18	7.83E-17
ZNF350-AS1	22.887	6.649	-1.783	0.001839641	0.00351972
AC073174.1	6.359	1.124	-2.5	7.03E-12	7.61E-11
CDKN2B	6.545	13.167	1.009	4.13E-05	0.000111152
NPIPB15	4.786	1.836	-1.382	5.84E-11	5.16E-10
LGALS7	1.549	5.467	1.82	0.000662191	0.001391575
CLIC3	30.897	73.156	1.243	6.45E-05	0.000166809
AC103691.1	4.425	1.84	-1.266	9.59E-21	1.62E-18
MOGAT2	1.753	0.584	-1.586	2.37E-12	2.90E-11
AKT3	1.682	3.503	1.058	6.69E-13	9.32E-12
FAM78B	2.665	0.754	-1.822	0.021792603	0.033022789

PLN	2.165	6.784	1.648	1.66E-09	1.09E-08
SPP1	86.789	211.268	1.283	9.47E-12	9.99E-11
PPBP	0.173	2.678	3.95	0.000897616	0.00183329
MAMDC2	0.69	1.738	1.333	9.74E-11	8.24E-10
ANXA1	60.286	133.634	1.148	3.46E-15	8.73E-14
NNMT	18.842	50.094	1.411	2.41E-17	1.15E-15
SUSD2	2.178	5.261	1.272	0.00048004	0.001036766
MROH2A	2.818	1.402	-1.007	1.28E-11	1.31E-10
PLEKHH1	3.464	1.323	-1.389	4.17E-17	1.88E-15
SLC14A1	24.222	7.749	-1.644	1.49E-14	3.10E-13
IL36G	0.956	4.295	2.168	0.006169053	0.010568323
SFRP2	36.149	100.895	1.481	4.38E-18	2.74E-16
NID2	1.346	3.432	1.351	1.33E-13	2.17E-12
AF131215.6	1.773	0.649	-1.45	5.14E-17	2.26E-15
MAP1A	0.76	1.964	1.37	4.43E-16	1.49E-14
AC008752.3	2.176	1.039	-1.067	1.88E-19	1.91E-17
FPR3	3.807	7.633	1.004	2.34E-12	2.87E-11
PKP1	16.644	52.429	1.655	6.45E-05	0.000166809
SPRR2A	14.66	53.184	1.859	2.51E-08	1.28E-07
MIR6784	2.414	0.569	-2.084	6.60E-16	2.05E-14
GREM1	2.578	6.239	1.275	5.41E-15	1.29E-13
IL6	2.533	8.052	1.669	4.94E-12	5.54E-11
SULT2A1	7.045	2.44	-1.53	1.54E-11	1.54E-10
KRT1	4.8	57.408	3.58	3.22E-07	1.31E-06
TRPS1	0.61	1.596	1.388	1.90E-16	7.16E-15
CYP3A5	6.471	1.935	-1.742	1.82E-14	3.69E-13
MS4A8	2.421	0.682	-1.828	2.20E-11	2.15E-10
MEDAG	1.985	5.708	1.524	5.35E-10	3.92E-09
HOXB-AS3	1.594	0.623	-1.356	1.41E-09	9.48E-09
ADAM19	3.069	7.976	1.378	6.67E-17	2.85E-15
CYP4F22	19.984	7.424	-1.429	2.37E-12	2.90E-11
MIR100HG	0.674	1.66	1.299	3.78E-12	4.37E-11

SLC22A3	0.796	2.053	1.366	9.24E-12	9.78E-11
FAM20A	1.15	2.888	1.329	2.11E-16	7.84E-15
WASH6P	14.807	5.941	-1.317	4.68E-08	2.26E-07
COPZ2	4.471	10.417	1.22	5.68E-18	3.35E-16
TGM1	2.193	10.347	2.238	0.000150527	0.000360677
SULF1	6.938	16.835	1.279	2.95E-16	1.05E-14
RGS2	12.636	31.225	1.305	5.73E-14	1.02E-12
ADAM23	0.777	2.213	1.51	5.09E-07	2.00E-06
KLK13	0.662	2.449	1.888	0.006632896	0.011285642
AL133370.1	4.097	1.537	-1.415	4.91E-09	2.91E-08
GATA3	118.979	53.253	-1.16	1.10E-18	8.19E-17
PM20D1	43.072	17.336	-1.313	1.39E-07	6.12E-07
OLFML3	5.52	18.312	1.73	1.57E-17	7.81E-16
PCSK9	0.969	2.386	1.3	2.13E-14	4.21E-13
COL14A1	2.753	6.676	1.278	7.48E-07	2.84E-06
CASQ2	0.971	3.423	1.818	2.58E-06	8.75E-06
ELOVL4	0.873	2.054	1.234	1.64E-12	2.10E-11
PGM5	1.598	3.903	1.288	0.02940173	0.043262688
AC005180.2	0.627	2.091	1.738	0.003092745	0.005635226
COL12A1	11.63	25.226	1.117	9.45E-13	1.28E-11
TGFB3	2.703	6.675	1.304	1.38E-20	2.13E-18
MFAP5	1.248	5.945	2.252	4.28E-20	5.31E-18
REN	5.319	1.4	-1.926	0.000328434	0.000735149
MYLK	2.668	7.638	1.517	1.54E-14	3.17E-13
CXCL6	2.684	5.627	1.068	0.000627994	0.001325063
CLMP	2.896	6.556	1.179	7.46E-17	3.14E-15
CLCA4	22.01	9.79	-1.169	1.71E-06	5.99E-06
ADGRF1	7.866	2.762	-1.51	6.28E-12	6.89E-11
CAV1	21.268	65.811	1.63	7.80E-19	6.11E-17
CAVIN2	2.395	5.085	1.086	0.006479738	0.011046725
LINC01564	2.152	1.012	-1.088	6.16E-09	3.56E-08
DACT1	1.208	3.03	1.326	4.94E-15	1.19E-13

CILP	1.805	4.782	1.406	6.00E-11	5.28E-10
GLT8D2	1.757	4.223	1.265	3.04E-16	1.08E-14
SORBS1	2.204	6.25	1.503	0.00019926	0.000466447
COL6A1	50.945	147.734	1.536	5.63E-21	1.09E-18
AC091492.1	1.371	0.635	-1.111	0.000486323	0.001048942
GLIPR1	1.944	4.315	1.15	2.06E-16	7.69E-15
IGKV5-2	1.335	4.19	1.65	0.004926312	0.008597909
SPRR2G	0.552	17.57	4.993	2.18E-06	7.51E-06
AHNAK2	1.687	6.979	2.048	2.02E-20	2.85E-18
MSC	4.282	9.541	1.156	9.65E-12	1.01E-10
TBX1	12.364	4.756	-1.378	3.53E-12	4.13E-11
MIR7152	2.048	0.729	-1.49	5.03E-08	2.41E-07
RAB31	11.289	24.416	1.113	2.62E-20	3.48E-18
DSG3	6.974	36.675	2.395	4.10E-08	2.01E-07
LINC01614	0.895	3.437	1.942	9.61E-21	1.62E-18
RGS20	0.671	1.641	1.29	2.34E-12	2.87E-11
LINC01615	1.12	3.336	1.574	5.83E-16	1.85E-14
AC103706.1	3.135	1.534	-1.032	9.42E-15	2.11E-13
ZNF682	2.494	1.175	-1.086	4.50E-16	1.50E-14
FGFR3	78.769	35.29	-1.158	4.21E-09	2.53E-08
SERPINB13	4.13	8.327	1.012	0.000153968	0.000368446
EMP3	10.884	29.137	1.421	8.48E-20	9.34E-18
HTRA1	36.693	77.776	1.084	2.02E-14	4.03E-13
MT-ND6	2962.892	1242.062	-1.254	2.36E-15	6.28E-14
AC133041.1	3.244	1.613	-1.008	3.27E-10	2.52E-09
EMP1	11.918	27.267	1.194	3.59E-17	1.64E-15
COLCA1	3.924	1.781	-1.139	1.53E-06	5.41E-06
MAOB	1.812	5.805	1.68	1.42E-13	2.31E-12
CTSK	28.966	74.603	1.365	1.40E-12	1.82E-11
IDUA	8.066	3.847	-1.068	8.82E-22	2.44E-19
VIPR1	4.888	2.337	-1.065	3.73E-12	4.32E-11
MIR3972	4.637	1.893	-1.293	4.56E-08	2.20E-07

AC090954.1	2.209	0.446	-2.309	2.48E-19	2.38E-17
RUNX2	1.606	3.411	1.087	1.24E-14	2.67E-13
PRSS1	10.586	3.019	-1.81	5.12E-05	0.000135063
GNB4	1.995	4.486	1.169	1.04E-21	2.70E-19
SERPINE2	2.166	6.335	1.548	1.25E-14	2.68E-13
ACP5	16.095	32.8	1.027	3.73E-06	1.23E-05
CYP1A1	82.673	26.86	-1.622	0.002057459	0.003897927
ECHDC3	1.356	2.905	1.099	3.82E-08	1.88E-07
COL15A1	7.852	15.734	1.003	6.25E-11	5.48E-10
RHOD	22.487	50.905	1.179	5.60E-09	3.28E-08
CSPG4	3.424	8.1	1.242	2.68E-10	2.10E-09
SLC38A5	5.25	10.802	1.041	2.48E-06	8.45E-06
RBP7	3.172	6.696	1.078	8.88E-07	3.31E-06
HES2	3.943	9.511	1.271	7.28E-09	4.15E-08
CLDN6	0.285	4.019	3.817	2.24E-07	9.47E-07
MT-CYB	7865.571	3806.092	-1.047	6.18E-18	3.55E-16
LINC00967	4.828	1.562	-1.629	4.24E-13	6.22E-12
TMTC1	0.664	1.383	1.059	9.28E-05	0.00023219
SLPI	206.043	470.875	1.192	0.000400206	0.000879237
APOL4	19.728	9.357	-1.076	5.69E-11	5.05E-10
DHRS2	203.037	80.128	-1.341	5.43E-16	1.76E-14
PDE10A	2.629	0.939	-1.485	1.05E-13	1.76E-12
CYP1A2	12.872	0.257	-5.644	0.006642125	0.011298389
TMEM158	4.476	11.624	1.377	2.50E-14	4.82E-13
IL17RD	0.671	1.385	1.044	1.26E-11	1.29E-10
AC012307.1	3.342	0.294	-3.505	2.27E-16	8.36E-15
GAS7	1.139	2.663	1.225	2.19E-14	4.29E-13
ABCA4	0.816	1.692	1.053	1.12E-05	3.37E-05
PXDN	3.817	10.358	1.44	2.33E-19	2.27E-17
PDGFC	1.829	3.684	1.01	3.50E-14	6.49E-13
TENT5B	1.745	5.908	1.76	4.57E-11	4.16E-10
CASP14	16.907	62.68	1.89	0.001314552	0.002588895

ITGAM	1.339	2.852	1.091	9.88E-13	1.33E-11
INHBA	3.396	8.151	1.263	5.54E-17	2.42E-15
FSTL1	20.12	40.642	1.014	3.59E-16	1.24E-14
AC010998.3	2.041	0.387	-2.401	1.75E-13	2.81E-12
PPP1R1B	8.6	4.167	-1.045	0.000721014	0.00150401
THY1	12.898	26.37	1.032	2.04E-12	2.55E-11
FCGR2A	4.194	8.627	1.04	3.81E-15	9.45E-14
ZNF626	3.805	1.797	-1.082	1.94E-05	5.58E-05
AC006042.1	4.327	1.548	-1.483	3.30E-20	4.16E-18
TNFRSF14-AS1	1.666	0.682	-1.29	1.80E-14	3.65E-13
FAM129A	3.691	8.626	1.225	2.33E-14	4.54E-13
MYOSLID	0.404	2.38	2.558	2.61E-12	3.16E-11
DEGS2	7.628	2.778	-1.457	1.19E-13	1.98E-12
CTGF	40.988	88.721	1.114	3.22E-12	3.81E-11
DOK7	2.219	0.985	-1.171	2.06E-12	2.58E-11
IGLC7	4.321	12.819	1.569	0.024071982	0.036135756
WISP2	0.927	2.231	1.266	5.48E-06	1.76E-05
GSTA2	1.658	0.582	-1.51	3.75E-05	0.000101854
AC026369.1	2.36	0.441	-2.418	3.30E-12	3.89E-11
AL450384.2	3.282	0.986	-1.735	9.44E-24	8.77E-21
PDGFRB	8.26	18.029	1.126	2.45E-15	6.44E-14
COMP	13.73	38.249	1.478	1.45E-09	9.74E-09
SMIM22	45.031	19.34	-1.219	1.86E-16	7.02E-15
C19orf18	1.747	0.825	-1.082	6.52E-12	7.13E-11
CMKLR1	1.305	2.677	1.037	1.34E-09	9.08E-09
CRTAC1	30.117	2.677	-3.492	1.46E-11	1.48E-10
PICSAR	2.696	5.969	1.147	7.65E-06	2.40E-05
MIR429	11.002	3.354	-1.714	1.16E-17	6.06E-16
TTR	12.851	1.335	-3.267	1.06E-10	8.91E-10
TEKT5	1.366	0.664	-1.039	1.82E-19	1.86E-17
DAB1	1.591	0.574	-1.472	0.000258699	0.000591949
MCF2L-AS1	4.667	2.107	-1.147	2.57E-13	3.94E-12

SRMS	4.555	2.221	-1.036	8.04E-15	1.84E-13
SULT2B1	2.698	6.527	1.275	2.87E-05	7.94E-05
CLEC11A	7.203	16.059	1.157	5.10E-09	3.01E-08
ACTA2	62.267	152.862	1.296	1.41E-09	9.47E-09
SPARC	153.23	337.31	1.138	1.97E-14	3.94E-13
EPHA3	0.671	1.359	1.017	2.09E-05	5.97E-05
ARSJ	0.65	1.443	1.15	6.42E-18	3.68E-16
NRP2	1.921	4.453	1.212	5.82E-21	1.10E-18
LGALS4	13.49	5.812	-1.215	5.42E-12	6.04E-11
TMEM178A	1.512	0.578	-1.387	1.53E-14	3.16E-13
AL139393.2	0.75	1.568	1.064	2.37E-12	2.90E-11
CAPN8	5.702	2.563	-1.154	1.08E-08	5.91E-08
TBX2	29.442	13.823	-1.091	2.91E-12	3.49E-11
TEAD4	5.215	10.822	1.053	3.23E-18	2.06E-16
TGFB2	0.747	1.663	1.154	2.36E-10	1.86E-09
COL1A2	110.557	316.489	1.517	3.98E-19	3.48E-17
MSN	30.563	66.217	1.115	3.89E-16	1.32E-14
FXYD6	1.476	4.2	1.509	3.06E-13	4.59E-12
ECM2	0.677	1.37	1.017	1.28E-11	1.31E-10
UBE2FP3	1.536	0.712	-1.109	8.16E-17	3.42E-15
OLFML2B	5.956	14.116	1.245	5.76E-15	1.36E-13
ERICH5	6.376	3.162	-1.012	7.70E-13	1.06E-11
RFLNA	0.577	1.991	1.788	2.46E-13	3.78E-12
SOST	0.982	4.007	2.029	3.37E-06	1.12E-05
HEPH	1.258	3.03	1.269	4.44E-12	5.04E-11
PTPRR	5.892	1.668	-1.821	1.79E-13	2.86E-12
MTCO3P12	55.728	12.429	-2.165	2.10E-12	2.62E-11
CHRDL1	0.899	2.357	1.39	1.08E-05	3.27E-05
EVA1A	1.188	3.132	1.398	3.23E-14	6.05E-13
LINC02178	0.439	5.186	3.564	0.002809895	0.005166901
ROR2	1.4	3.306	1.24	3.67E-17	1.68E-15
COL3A1	175.971	523.781	1.574	1.15E-17	6.02E-16

CDH17	0.195	2.773	3.832	0.011073358	0.017917405
AL391834.2	4.758	2.308	-1.044	5.59E-18	3.32E-16
INTS6L	2.954	1.423	-1.054	1.87E-15	5.18E-14
EFEMP1	7.317	25.075	1.777	1.20E-20	1.97E-18
RF02247	1.75	0.391	-2.161	1.94E-09	1.26E-08
CYP4Z2P	1.626	0.533	-1.609	8.71E-18	4.80E-16
PRSS2	59.192	9.139	-2.695	0.001205862	0.002392228
AC018695.6	3.828	1.694	-1.176	6.89E-12	7.49E-11
MAP1B	1.506	4.349	1.53	1.20E-16	4.79E-15
CNTN1	0.68	3.394	2.318	1.10E-14	2.42E-13
TNFAIP8L3	0.928	2.662	1.52	2.20E-21	5.02E-19
MYH11	13.008	50.668	1.962	0.00120788	0.002394769
GOLGA8B	4.172	1.954	-1.094	1.40E-12	1.82E-11
CYP4F8	17.615	3.399	-2.374	4.92E-14	8.88E-13
RNASE2	0.65	1.735	1.417	1.86E-13	2.95E-12
PSORS1C3	6.285	2.072	-1.601	7.48E-09	4.24E-08
GPR176	1.423	3.518	1.306	1.26E-19	1.36E-17
TRBV30	2.296	0.944	-1.283	0.001333372	0.002621594
NADSYN1	11.62	5.627	-1.046	5.88E-27	2.55E-23
DSE	1.602	3.302	1.043	3.14E-17	1.46E-15
AC010487.2	3.46	1.298	-1.414	3.86E-16	1.32E-14
MIR3189	12.003	5.882	-1.029	2.07E-12	2.58E-11
FAM126A	0.973	2.029	1.061	4.58E-18	2.82E-16
NELL2	0.889	2.74	1.624	0.007689945	0.012871999
RPL37P1	1.952	0.72	-1.439	1.60E-23	1.23E-20
NTN1	0.84	2.464	1.553	1.90E-14	3.83E-13
NEIL1	3.962	1.814	-1.127	1.32E-20	2.09E-18
PTHLH	4.892	21.077	2.107	2.76E-11	2.64E-10
MT-TT	8.248	3.163	-1.383	1.54E-09	1.03E-08
KIRREL1	2.351	5.947	1.339	1.21E-20	1.97E-18
CHST15	2.231	5.361	1.264	6.11E-22	1.85E-19
SULT1A2	1.851	0.919	-1.01	4.23E-09	2.54E-08

SIGLEC5	0.901	2.724	1.596	0.028181253	0.041623581
TWIST2	0.788	2.327	1.563	5.97E-14	1.05E-12
RHEX	4.496	1.65	-1.447	9.53E-05	0.000237944
AL691482.3	12.836	3.874	-1.728	5.15E-22	1.61E-19
NKILA	0.707	1.86	1.396	1.06E-16	4.31E-15
SPINK4	10.108	1.698	-2.574	3.63E-10	2.78E-09
OSMR	5.097	12.397	1.282	5.81E-18	3.39E-16
CNGA1	6.209	2.917	-1.09	6.72E-12	7.34E-11
RARRES2	21.533	45.119	1.067	2.90E-08	1.45E-07
POPDC2	1.259	2.962	1.234	0.017644748	0.027306949
SCNN1G	22.783	8.46	-1.429	1.09E-14	2.40E-13
CDH26	4.387	1.921	-1.192	1.12E-11	1.16E-10
SUGCT	0.801	2.668	1.736	2.05E-16	7.66E-15
STRA6	1.457	3.25	1.158	0.000116898	0.000286539
AC110285.2	2.842	1.419	-1.002	4.82E-12	5.43E-11
LRRC17	0.922	2.311	1.326	1.59E-09	1.06E-08
AL158206.1	88.173	33.202	-1.409	5.02E-18	3.05E-16
COL5A2	16.608	45.798	1.463	5.08E-20	5.90E-18
LYVE1	0.613	2.853	2.219	5.40E-08	2.57E-07
DDR2	1.269	2.853	1.168	4.18E-12	4.77E-11
SLC38A4	6.5	2.101	-1.629	0.002934945	0.005374078
ATP1A4	2.548	0.949	-1.425	5.46E-13	7.78E-12
LEAP2	3.849	0.907	-2.086	6.99E-21	1.30E-18
PAN2	9.633	4.669	-1.045	1.52E-15	4.31E-14
KRT6C	3.39	32.07	3.242	3.86E-07	1.56E-06
FHL1	3.74	11.025	1.56	7.36E-08	3.41E-07
LUM	70.153	156.185	1.155	6.85E-12	7.45E-11
AOC2	2.613	0.919	-1.507	4.58E-18	2.82E-16
ACOXL	2.992	0.992	-1.593	5.81E-18	3.39E-16
CAPS	45.292	21.517	-1.074	3.00E-14	5.68E-13
DCBLD2	3.266	7.618	1.222	4.50E-10	3.35E-09
KRTAP5-9	3.034	0.92	-1.722	1.29E-16	5.04E-15

KCNMB1	0.845	2.256	1.418	1.21E-08	6.56E-08
PDCL3P4	1.911	0.861	-1.149	1.16E-17	6.04E-16
KRT14	204.099	705.745	1.79	1.38E-05	4.09E-05
CD163	4.853	11.597	1.257	2.43E-15	6.41E-14
CYTL1	0.78	2.622	1.749	3.52E-07	1.43E-06
SNORD123	6.342	2.419	-1.391	1.37E-17	6.93E-16
SORCS2	0.689	1.532	1.153	1.89E-10	1.52E-09
COL5A3	2.839	7.605	1.422	1.84E-20	2.63E-18
COL5A1	15.842	47.332	1.579	2.85E-20	3.67E-18
PLAU	45.074	104.651	1.215	2.86E-15	7.42E-14
NRCAM	0.883	2.04	1.209	4.23E-06	1.38E-05
TNFAIP6	1.836	5.39	1.554	3.32E-21	7.21E-19
CRACR2B	14.109	5.551	-1.346	1.58E-19	1.65E-17
OGN	0.566	1.811	1.678	1.21E-07	5.39E-07
ITPKA	0.794	1.69	1.09	1.39E-05	4.12E-05
CYP4F12	11.393	2.962	-1.943	5.02E-20	5.88E-18
PCP4	3.668	18.7	2.35	0.005497189	0.009516364
CYP4F23P	9.328	2.906	-1.683	2.48E-18	1.67E-16
MT1X	18.585	44.892	1.272	2.08E-20	2.91E-18
CRISPLD2	4.969	11.297	1.185	1.20E-13	1.99E-12
WNT5B	2.467	5.854	1.247	2.86E-07	1.18E-06
CSF3R	1.208	2.663	1.14	1.12E-05	3.37E-05
SHROOM1	15.752	6.415	-1.296	2.70E-16	9.72E-15
CD70	0.765	2.266	1.566	5.58E-08	2.65E-07
AL359715.1	1.542	0.531	-1.538	5.51E-19	4.51E-17
AL023284.4	11.512	5.613	-1.036	3.38E-12	3.98E-11
FERMT2	2.469	5.256	1.09	2.40E-15	6.35E-14
KRT5	280.786	852.74	1.603	1.04E-06	3.83E-06
CYBRD1	6.426	13.833	1.106	3.70E-16	1.27E-14
P4HA3	0.745	2.095	1.491	1.69E-21	4.08E-19
POSTN	21.864	63.84	1.546	1.49E-19	1.58E-17
CWH43	1.185	2.589	1.128	0.021615371	0.032792432

IHH	2.875	1.041	-1.466	2.41E-07	1.01E-06
AP005432.2	26.266	6.078	-2.111	4.20E-07	1.68E-06
MTATP8P2	21.5	7.405	-1.538	7.04E-08	3.27E-07
IGF2BP2	3.965	9.168	1.209	1.56E-14	3.21E-13
IL1RAP	1.555	3.158	1.023	6.89E-12	7.49E-11
WISP1	1.273	4.431	1.799	9.70E-18	5.26E-16
CTSE	36.613	9.774	-1.905	4.00E-10	3.02E-09
ADAMTS15	0.724	2.009	1.472	1.17E-08	6.38E-08
RAB23	1.445	3.074	1.089	2.37E-17	1.14E-15
MELTF	2.961	6.279	1.085	2.88E-13	4.37E-12
TNNI2	57.842	21.088	-1.456	2.47E-08	1.26E-07
SMOC2	4.84	10.073	1.058	0.000385807	0.000850044
ST3GAL5	11.405	4.998	-1.19	2.05E-15	5.62E-14
KRT34	0.215	1.789	3.055	1.79E-09	1.17E-08
KRT16	82.688	340.596	2.042	4.26E-08	2.08E-07
CCDC80	3.016	10.358	1.78	1.74E-20	2.57E-18
UPK3A	111.467	47.428	-1.233	2.31E-08	1.19E-07
GRP	0.487	1.594	1.711	9.92E-08	4.51E-07
TOX2	1.408	3.386	1.266	3.49E-05	9.53E-05
S100A7	85.404	489.367	2.519	2.32E-06	7.94E-06
TNFAIP2	164.426	81.117	-1.019	1.20E-15	3.49E-14
LAMA3	4.942	15.145	1.616	9.96E-06	3.05E-05
TPD52L1	3.099	6.198	1	1.11E-11	1.16E-10
PPP2R2C	0.512	1.595	1.64	1.29E-05	3.85E-05
PGLYRP4	0.809	3.352	2.05	0.000566464	0.001205799
SPOCD1	8.437	2.639	-1.677	2.75E-07	1.14E-06
LINC01764	2.755	0.493	-2.483	3.88E-09	2.36E-08
CDA	5.113	17.903	1.808	2.45E-14	4.73E-13
TGFBI	18.191	72.052	1.986	8.03E-22	2.27E-19
AL772337.3	1.889	0.576	-1.713	4.88E-11	4.39E-10
PTX3	1.493	5.061	1.762	1.29E-12	1.69E-11
BTNL9	1.657	0.736	-1.171	3.04E-08	1.51E-07

AL022322.1	1.926	0.821	-1.23	4.47E-18	2.78E-16
CYP26B1	0.651	3.092	2.249	2.70E-12	3.26E-11
DSC2	3.4	11.589	1.769	1.07E-13	1.80E-12
AXL	5.646	14.633	1.374	1.94E-15	5.35E-14
MIR200B	2.11	0.614	-1.782	2.64E-11	2.53E-10
COL10A1	3.748	10.392	1.471	3.62E-16	1.24E-14
FAM174B	26.455	11.6	-1.189	3.55E-15	8.87E-14
ALKAL1	1.733	0.856	-1.017	2.27E-09	1.46E-08
IL9R	2.023	0.753	-1.425	1.29E-14	2.74E-13
AP000424.1	1.843	0.466	-1.983	6.07E-13	8.53E-12
CD96	3.508	1.676	-1.066	2.93E-10	2.28E-09
IGLV4-60	2.472	9.386	1.925	0.008520906	0.01413197
SMOC1	0.812	2.447	1.59	0.000869536	0.001780975
KLK6	8.906	19.864	1.157	1.85E-12	2.34E-11
KLHDC7A	9.528	4.496	-1.084	3.99E-14	7.26E-13
SPINK1	562.964	123.042	-2.194	5.47E-19	4.50E-17
FAM20C	4.312	11.158	1.372	2.55E-19	2.42E-17
RF02246	6.596	1.554	-2.085	2.56E-09	1.62E-08
NEXN	1.82	3.993	1.134	2.43E-12	2.96E-11
CD14	25.685	53.7	1.064	2.45E-14	4.73E-13
PHGR1	7.704	1.986	-1.956	2.34E-15	6.23E-14
S100A7A	1.292	5.179	2.004	1.99E-06	6.90E-06
RHOXF1-AS1	7.523	3.146	-1.258	0.000255452	0.000584931
TUBB6	13.823	35.067	1.343	3.19E-21	7.03E-19
LINC02544	0.412	1.59	1.949	1.07E-15	3.17E-14
AL390719.2	12.757	4.133	-1.626	2.04E-23	1.48E-20
MTRNR2L12	2.98	1.08	-1.465	5.62E-12	6.25E-11
KCNE4	0.916	2.355	1.363	3.51E-12	4.11E-11
AL390294.1	3.696	1.102	-1.746	3.22E-19	2.87E-17
RNF128	39.024	16.55	-1.238	2.52E-15	6.59E-14
PDZRN3	1.147	2.699	1.234	2.69E-08	1.36E-07
MIR6730	1.401	0.619	-1.177	1.30E-08	6.99E-08

BGN	111.661	242.182	1.117	1.46E-14	3.04E-13
PRR9	1.984	11.32	2.512	0.026564953	0.039504206
F3	18.11	39.676	1.131	5.82E-11	5.15E-10
TESC	20.779	7.795	-1.414	7.28E-11	6.30E-10
CD244	0.533	1.861	1.803	0.007432825	0.012488241
KRT6B	21.343	136.961	2.682	5.35E-10	3.92E-09
AP002026.1	1.751	0.611	-1.519	3.18E-18	2.04E-16
P2RX1	0.733	2.383	1.701	5.68E-08	2.69E-07
CCL13	4.252	8.571	1.011	2.60E-06	8.82E-06
C5orf46	0.926	1.94	1.067	2.62E-11	2.52E-10
PNCK	8.272	3.249	-1.348	2.00E-06	6.93E-06
EMILIN1	13.433	33.458	1.317	1.36E-13	2.23E-12
MYBPH	0.042	3.612	6.417	1.10E-05	3.32E-05
RAET1L	0.511	1.98	1.955	6.26E-11	5.48E-10
DIO2	1.36	3.002	1.143	1.59E-09	1.06E-08
TRPV2	2.95	6.206	1.073	1.06E-08	5.82E-08
SPHK1	4.848	12.622	1.38	7.11E-23	3.51E-20
CFL2	1.79	3.714	1.053	9.41E-18	5.12E-16
TXLNGY	2.574	1.256	-1.035	5.64E-07	2.19E-06
LTBP2	6.542	14.74	1.172	1.29E-14	2.74E-13
FMO8P	1.735	0.342	-2.343	2.99E-14	5.68E-13
RAB3IL1	2.055	4.248	1.047	4.57E-15	1.11E-13
ACER2	21.748	10.283	-1.081	1.27E-10	1.05E-09
ISLR	16.762	46.298	1.466	4.67E-15	1.13E-13
PPARG	46.905	19.882	-1.238	2.35E-19	2.27E-17
ZIC2	1.165	2.508	1.107	0.000543526	0.001160771
BARX2	1.871	6.611	1.821	1.44E-06	5.14E-06
PTGIS	3.484	9.614	1.464	5.04E-08	2.42E-07
MMP11	22.46	52.536	1.226	1.96E-12	2.46E-11
NBPF11	1.962	0.972	-1.014	2.50E-17	1.18E-15
MT-TM	4.012	1.177	-1.769	3.79E-10	2.88E-09
SERPINF1	20.298	47.978	1.241	2.74E-11	2.62E-10

TPSAB1	3.484	7.918	1.184	1.98E-07	8.47E-07
FMO9P	15.443	7.34	-1.073	2.70E-13	4.11E-12
HEPHL1	0.69	2.181	1.661	0.000104137	0.00025813
FPR1	1.481	3.947	1.414	2.35E-16	8.60E-15
MMP9	12.797	86.563	2.758	1.90E-14	3.83E-13
MXRA5	7.423	16.375	1.141	7.86E-10	5.57E-09
TBX2-AS1	13.123	5.634	-1.22	1.51E-13	2.45E-12
PPM1N	5.008	1.536	-1.705	2.16E-13	3.39E-12
AC091544.4	2.158	0.443	-2.285	1.33E-22	5.08E-20
IBSP	0.488	3.368	2.788	4.11E-06	1.35E-05
AC068594.1	2.841	0.962	-1.562	6.55E-14	1.15E-12
LOX	2.57	7.724	1.588	4.66E-24	5.05E-21
HTRA3	12.989	30.563	1.234	8.58E-18	4.77E-16
LINC00930	6.585	0.721	-3.192	4.57E-21	9.14E-19
IGFBP6	18.046	46.248	1.358	7.59E-07	2.87E-06
EPGN	0.618	2.124	1.781	0.000121705	0.000297312
MMP2	56.447	123.159	1.126	3.32E-11	3.10E-10
CHRDL2	1.957	7.096	1.858	2.59E-13	3.97E-12
MEG3	0.787	2.256	1.519	0.000157042	0.00037525
ITGBL1	0.699	1.702	1.285	4.14E-11	3.80E-10
DCN	12.006	31.5	1.392	9.27E-13	1.26E-11
ADAMTSL4	3.707	7.813	1.076	7.46E-13	1.03E-11
TRNP1	21.837	8.568	-1.35	4.01E-07	1.61E-06
RPLP0P2	0.583	1.493	1.357	4.40E-16	1.48E-14
FER1L4	46.27	11.955	-1.953	1.17E-28	1.52E-24
HAS1	0.398	1.837	2.207	7.02E-11	6.09E-10
ACP7	0.591	1.543	1.384	0.001769422	0.003397905
CSF2	1.039	2.165	1.059	0.012929861	0.020590887
GNG4	1.14	2.936	1.365	3.70E-14	6.79E-13
GAS6	10.85	25.675	1.243	6.39E-13	8.94E-12
UPK2	460.622	215.476	-1.096	5.57E-10	4.06E-09
MTND4P12	86.169	36.895	-1.224	1.45E-07	6.39E-07

CCNA1	0.271	2.532	3.224	1.39E-09	9.38E-09
CCL15	2.866	0.875	-1.712	3.44E-14	6.39E-13
S100A9	1214.255	3433.943	1.5	2.66E-07	1.11E-06
AC079466.1	2.264	0.132	-4.103	1.86E-09	1.21E-08
GPR68	2.563	7.315	1.513	8.01E-16	2.46E-14
GPT	1.473	0.698	-1.077	2.63E-11	2.53E-10
RALGAPA2	11.321	5.487	-1.045	9.36E-12	9.88E-11
CAVIN1	40.635	96.223	1.244	2.39E-21	5.37E-19
SLC2A3	4.445	11.792	1.407	2.08E-13	3.27E-12
TENM2	0.592	2.784	2.233	6.01E-12	6.65E-11
SULT1E1	8.487	2.942	-1.528	0.0004802	0.00103694
BTBD16	29.949	6.14	-2.286	1.55E-17	7.78E-16
RNU5B-2P	3.673	1.826	-1.009	1.19E-15	3.46E-14
COL11A1	2.058	7.631	1.891	2.68E-12	3.24E-11
ANGPTL1	0.394	1.754	2.154	1.19E-05	3.58E-05
SPINK6	0.956	3.063	1.68	0.000134829	0.000326252
PDLIM3	2.466	6.498	1.398	2.94E-15	7.60E-14
MIR548AN	1.751	0.563	-1.638	4.84E-11	4.37E-10
ZNF737	7.357	2.883	-1.352	1.13E-10	9.49E-10
S100A8	233.194	1023.548	2.134	4.35E-10	3.25E-09
ERN2	5.537	1.743	-1.668	1.52E-09	1.02E-08
AC112484.3	1.423	0.677	-1.071	6.22E-10	4.50E-09
WNT11	1.159	3.662	1.66	2.72E-12	3.28E-11
THBD	18.139	38.54	1.087	2.65E-06	8.97E-06
BOC	0.539	1.766	1.712	3.30E-14	6.16E-13
HMGCS2	156.458	36.447	-2.102	8.17E-15	1.87E-13
SPRR2D	6.594	27.796	2.076	1.21E-10	1.01E-09
RHBG	6.34	2.002	-1.663	1.33E-14	2.80E-13
CD44	28.599	58.004	1.02	3.28E-12	3.87E-11
FNDC1	1.552	4.782	1.623	1.12E-16	4.53E-15
ADAM12	1.906	4.944	1.375	4.60E-17	2.06E-15
BICDL2	20.144	9.085	-1.149	3.24E-19	2.87E-17

GGT6	31.736	14.315	-1.149	5.57E-16	1.78E-14
TCF7L1	2.685	5.463	1.025	1.56E-15	4.40E-14
FBN2	1.779	4.004	1.171	9.66E-08	4.40E-07
AC002059.1	2.568	1.022	-1.33	2.13E-15	5.75E-14
CSDC2	0.727	1.961	1.431	1.12E-11	1.17E-10
TPM1	10.191	23.494	1.205	4.74E-19	3.98E-17
CCL24	0.707	2.301	1.702	2.11E-09	1.36E-08
EIF4HP2	2.06	0.898	-1.198	3.11E-20	3.97E-18
CXCL5	1.808	8.079	2.16	4.09E-09	2.46E-08
SIGLEC15	3.745	1.454	-1.365	6.93E-16	2.15E-14
PGLYRP3	0.878	4.647	2.405	2.24E-05	6.35E-05
PLCH2	5.612	2.69	-1.061	2.92E-07	1.20E-06
AL158847.1	2.694	1.038	-1.376	4.04E-05	0.000108896
ME1	4.206	8.588	1.03	8.72E-17	3.63E-15
CFAP53	1.674	0.781	-1.101	3.43E-14	6.39E-13
RNU6ATAC18P	1.962	0.976	-1.007	6.20E-10	4.48E-09
LMCD1	2.385	5.304	1.153	3.22E-13	4.82E-12
UPK3BL1	1.475	0.671	-1.136	0.017312375	0.026814904
AL139280.1	1.557	0.692	-1.169	0.003357055	0.006075961
TPM2	35.931	89.546	1.317	2.98E-11	2.83E-10
ATP8B2	1.738	3.782	1.122	2.98E-13	4.49E-12
AL161669.3	4.37	1.986	-1.138	5.36E-05	0.000140678
DEGS1	14.73	32.122	1.125	4.91E-26	1.28E-22
KLK7	1.185	6.525	2.46	3.55E-12	4.14E-11
S100P	769.609	360.366	-1.095	1.11E-12	1.47E-11
AREG	16.942	36.776	1.118	8.39E-06	2.61E-05
CD109	2.265	7.37	1.702	1.05E-25	2.27E-22
PDGFRL	1.911	3.859	1.014	3.93E-09	2.38E-08
PRELP	2.613	5.516	1.078	2.86E-05	7.91E-05
GPC3	17.792	4.881	-1.866	0.000683924	0.001433078
MAGIX	3.548	1.422	-1.319	2.72E-16	9.76E-15
FGFBP1	13.706	62.597	2.191	1.97E-06	6.82E-06

PRR16	0.681	1.798	1.4	7.68E-19	6.05E-17
MT-ND5	2369.208	983.35	-1.269	1.97E-17	9.65E-16
FKBP10	19.165	42.728	1.157	7.29E-14	1.26E-12
S100A5	3.676	1.468	-1.324	3.93E-15	9.71E-14
TUBB2A	8.89	18.05	1.022	1.70E-14	3.47E-13
COL8A1	3.177	6.682	1.072	1.61E-09	1.07E-08
FLNA	59.18	147.562	1.318	7.57E-22	2.22E-19
SNORD69	3.745	1.541	-1.281	2.05E-17	1.00E-15
IL36RN	0.681	2.683	1.978	1.85E-05	5.33E-05
MIR200A	3.396	1.007	-1.754	8.58E-16	2.61E-14
ISM1	0.995	2.061	1.05	0.000574535	0.00122098
VCAN	6.259	14.215	1.183	1.50E-15	4.26E-14
SCARF2	2.163	5.256	1.281	3.25E-14	6.09E-13
CAVIN3	8.355	17.161	1.038	5.47E-16	1.77E-14
DPT	3.139	9.55	1.605	2.40E-15	6.35E-14
LAMA2	1.067	2.184	1.033	3.76E-10	2.86E-09
MT1A	1.335	5.76	2.109	3.41E-09	2.10E-08
SLC44A4	30.068	14.364	-1.066	2.46E-11	2.38E-10
PRRX1	2.355	5.306	1.172	7.35E-21	1.35E-18
NPNT	2.535	5.524	1.124	6.61E-08	3.10E-07
CAPN9	2.431	0.648	-1.907	6.58E-10	4.73E-09
ADAMTS14	1.092	2.665	1.287	1.10E-15	3.23E-14
RPL29P19	2.037	6.224	1.611	5.84E-13	8.24E-12
TAGLN	54.764	140.173	1.356	2.78E-11	2.65E-10
HOXB6	7.944	3.526	-1.172	2.08E-08	1.08E-07
CTSG	0.766	2.953	1.947	4.10E-06	1.35E-05
AC004148.2	3.068	1.438	-1.094	5.83E-24	5.83E-21
CORO6	0.881	1.972	1.163	2.41E-07	1.01E-06
EDNRA	1.779	3.762	1.08	1.17E-13	1.96E-12
ADAM33	0.649	1.474	1.185	0.000316493	0.000711056
HOXB8	7.227	3.355	-1.107	1.22E-05	3.66E-05
SYNPO2	2.298	7.766	1.757	0.002383987	0.004454265

HSD17B2	8.283	2.765	-1.583	6.13E-14	1.08E-12
AEBP1	37.965	92.1	1.279	4.99E-17	2.22E-15
CALD1	13.322	31.657	1.249	3.71E-21	7.90E-19
TOX3	8.439	2.757	-1.614	8.83E-15	1.99E-13
SLC25A27	1.787	0.851	-1.07	4.31E-08	2.09E-07
VSIG4	5.305	12.189	1.2	1.16E-14	2.53E-13
MSLN	4.517	13.129	1.539	0.00017238	0.000409045
CD209	1.168	3.232	1.468	3.83E-12	4.41E-11
GPX8	4.215	8.758	1.055	2.09E-17	1.02E-15
AC105219.1	2.46	0.908	-1.438	2.78E-11	2.65E-10
TGFB1I1	4.145	8.759	1.079	1.04E-13	1.75E-12
PAPSS2	2.714	5.524	1.025	2.17E-16	8.03E-15
MUC2	2.684	1.088	-1.303	1.89E-08	9.87E-08
STXBP1	1.548	3.117	1.01	1.10E-16	4.44E-15
C6orf15	0.18	6.787	5.233	2.68E-12	3.24E-11
CAV2	6.894	14.944	1.116	3.29E-15	8.42E-14
LINC01451	2.613	0.87	-1.587	7.79E-10	5.53E-09
PAQR6	3.938	1.772	-1.152	9.46E-14	1.60E-12
GPC6	1.012	2.653	1.39	1.20E-16	4.79E-15
SNAI1	1.895	4.369	1.205	8.91E-16	2.69E-14
FGF19	0.143	2.811	4.296	0.016246347	0.02531145
CGB8	0.13	2.153	4.051	0.000473819	0.001024861
PADI2	0.975	2.282	1.227	2.62E-09	1.66E-08
FAM3B	22.512	9.616	-1.227	1.63E-14	3.34E-13
STK32A	1.363	0.65	-1.069	2.93E-11	2.78E-10
CALML5	44.743	118.878	1.41	5.53E-07	2.15E-06
PI3	73.387	886.979	3.595	1.62E-09	1.07E-08
BHMT	25.379	6.273	-2.016	1.26E-15	3.64E-14
KATNAL1	0.689	1.624	1.237	1.17E-22	4.75E-20
AP000892.3	0.831	2.196	1.401	2.52E-08	1.28E-07
AC019117.1	9.814	1.998	-2.297	6.41E-19	5.18E-17
AC008105.2	2.518	1.04	-1.276	1.04E-18	7.83E-17

TNS1	4.542	10.626	1.226	2.30E-07	9.70E-07
TNC	7.347	28.329	1.947	2.82E-22	9.42E-20
ITGA5	10.751	30.543	1.506	2.42E-22	8.28E-20
FILIP1L	4.043	8.375	1.051	2.91E-12	3.49E-11
AC025575.2	6.557	3.117	-1.073	0.000801789	0.0016563
DNASE1L3	0.395	1.607	2.025	2.27E-06	7.78E-06
MTND1P23	66.365	18.503	-1.843	1.93E-09	1.25E-08
MRC1	2.556	5.429	1.087	3.71E-12	4.30E-11
AR	1.95	0.954	-1.031	1.20E-05	3.62E-05
FAP	1.239	3.542	1.516	1.77E-20	2.58E-18
F2RL2	0.564	1.518	1.428	2.89E-06	9.74E-06
CYTOR	3.092	6.805	1.138	3.04E-16	1.08E-14
VSIG2	131.583	42.604	-1.627	2.78E-19	2.55E-17
PCP2	2.811	1.159	-1.278	1.45E-18	1.05E-16
CCDC8	1.63	3.774	1.211	2.17E-12	2.69E-11
SGCA	0.852	2.196	1.366	0.000591548	0.001252852
RASD1	3.364	8.361	1.313	0.014345081	0.022606583
DSC3	11.885	34.292	1.529	1.00E-06	3.70E-06
DUOXA2	9.553	2.978	-1.682	3.45E-05	9.43E-05
TMEM51-AS1	1.968	0.686	-1.521	2.16E-18	1.51E-16
AC010329.1	2.491	0.745	-1.741	4.49E-11	4.08E-10
PYROXD2	4.141	2.035	-1.025	4.37E-13	6.39E-12
DES	70.333	288.19	2.035	3.42E-08	1.70E-07
GRAMD2A	0.578	1.78	1.623	0.000316493	0.000711056
STK32A-AS1	1.533	0.489	-1.649	5.15E-09	3.04E-08
PLA2G2F	23.603	10.913	-1.113	4.50E-13	6.55E-12
KLK8	0.97	5.028	2.374	4.62E-09	2.76E-08
GOLGA8A	10.117	4.358	-1.215	6.50E-16	2.03E-14
PMEPA1	11.956	27.868	1.221	7.35E-17	3.10E-15
NES	5.411	12.033	1.153	6.09E-10	4.41E-09
SCEL	1.19	4.992	2.068	1.40E-07	6.17E-07
OVGP1	3.958	1.168	-1.76	9.49E-23	4.11E-20

UCA1	81.997	32.757	-1.324	5.35E-07	2.09E-06
DSP	34.407	89.312	1.376	7.80E-13	1.07E-11
RCN3	14.423	34.473	1.257	7.93E-15	1.82E-13
PLPP4	1.463	3.674	1.329	8.37E-17	3.50E-15
SH3BP5-AS1	2.233	1.052	-1.086	1.41E-19	1.51E-17
ZBED2	1.847	4.335	1.231	7.74E-12	8.30E-11
AC108134.1	1.996	0.849	-1.234	2.92E-18	1.89E-16
SYTL5	3.613	1.589	-1.185	1.41E-08	7.54E-08
RASL10A	1.471	0.656	-1.164	4.63E-10	3.44E-09
MT-TY	9.484	4.669	-1.022	5.83E-09	3.39E-08
IGHV1OR15-2	0.666	1.343	1.012	0.002519454	0.004685832
RF00569	3.825	1.68	-1.187	3.00E-16	1.07E-14
MMP13	10.872	23.685	1.123	0.001142644	0.00227658
PAGE2B	3.492	1.276	-1.452	0.000225723	0.000522097
MYL9	63.918	168.734	1.4	1.06E-10	8.89E-10
SMAD6	2.564	1.184	-1.115	3.46E-14	6.41E-13
DUOX2	11.597	4.763	-1.284	1.02E-06	3.75E-06
BAG2	1.451	3.1	1.095	2.01E-19	2.01E-17
SYNM	2.831	11.477	2.019	2.39E-10	1.88E-09
AC068831.6	1.988	0.978	-1.023	2.57E-12	3.12E-11
FGF7	0.82	2.501	1.608	4.88E-11	4.39E-10
LRRC15	1.376	5.047	1.875	8.91E-16	2.69E-14
CAPN5	17.752	8.871	-1.001	4.38E-14	7.93E-13
PEG10	4.43	11.228	1.342	0.011573242	0.018638137
ZNF300	3.438	1.677	-1.036	2.90E-06	9.78E-06
RASD2	1.663	3.39	1.028	1.07E-05	3.24E-05
GKN1	2.098	0.385	-2.445	1.88E-07	8.06E-07
ADCY10P1	1.705	0.776	-1.136	6.15E-17	2.67E-15
VSNL1	1.648	4.016	1.285	2.71E-09	1.70E-08
SFTPA2	1.891	0.838	-1.174	7.16E-07	2.72E-06
NSG1	7.594	3.412	-1.155	0.000227283	0.000525052
PDPN	7.305	18.938	1.374	5.58E-13	7.90E-12

AC087741.1	2.064	1.024	-1.012	2.88E-18	1.87E-16
ALB	5.251	0.946	-2.473	0.00792862	0.013228945
PKDCC	1.43	3.832	1.422	5.91E-09	3.43E-08
IL11	1.305	4.137	1.664	3.55E-08	1.75E-07
SPRR1B	36.967	213.952	2.533	1.67E-07	7.26E-07
JPH2	1.188	2.926	1.301	6.84E-09	3.92E-08
PODNL1	1.179	3.164	1.424	8.65E-18	4.79E-16
LY6G6C	1.943	4.649	1.259	0.007488188	0.012574742
RBPMS2	2.563	5.379	1.069	4.31E-05	0.000115625
TRH	0.013	2.323	7.499	0.006182487	0.010585759
MFAP3L	4.118	1.983	-1.054	2.34E-13	3.62E-12
TUBB2B	3.315	9.149	1.465	2.48E-06	8.45E-06
ARHGEF4	1.26	2.834	1.17	1.32E-06	4.74E-06
CPA3	2.53	6.253	1.306	2.34E-08	1.20E-07
FGFR1	1.921	5.854	1.608	9.95E-13	1.33E-11
AC053503.4	0.464	1.775	1.935	3.82E-05	0.000103585
DPP4	0.861	3.343	1.957	8.07E-18	4.57E-16
GFPT2	1.248	5.652	2.179	6.36E-25	9.19E-22
SPON2	5.855	13.03	1.154	1.86E-16	7.02E-15
PTPRZ1	0.49	2.163	2.142	1.74E-09	1.14E-08
CASQ1	7.207	2.11	-1.772	2.05E-12	2.57E-11
ACTN1	19.483	39.118	1.006	2.71E-23	1.68E-20
PDGFRA	1.751	3.636	1.054	3.84E-08	1.89E-07
GEM	4.135	8.676	1.069	1.69E-11	1.68E-10
AL121790.2	2.698	0.996	-1.438	1.51E-10	1.24E-09
AMTN	0.572	2.986	2.385	0.005930277	0.010192835
OLR1	5.618	13.05	1.216	2.34E-05	6.61E-05
LINC01833	2.113	0.916	-1.207	6.17E-07	2.37E-06
AGR2	94.16	45.764	-1.041	3.69E-12	4.28E-11
CHST11	3.556	8.623	1.278	1.58E-24	2.05E-21
CLIC4	18.356	41.501	1.177	1.28E-26	4.16E-23
CTHRC1	17.68	46.89	1.407	7.70E-22	2.22E-19

STEAP4	1.228	3.293	1.423	1.17E-09	8.06E-09
TTLL3	3.527	1.476	-1.257	1.23E-22	4.85E-20
TIMP2	25.305	60.942	1.268	3.74E-18	2.35E-16
AC007490.1	2.083	0.644	-1.694	1.14E-16	4.60E-15
CRLF1	1.345	3.528	1.391	0.011954608	0.019176324
PCOLCE	12.019	30.492	1.343	4.18E-11	3.84E-10
CEMIP	1.967	4.497	1.193	1.82E-06	6.35E-06
SFRP1	1.806	4.897	1.439	1.53E-07	6.70E-07
DACT3	0.811	1.911	1.237	2.71E-09	1.70E-08
DNAJB5	1.252	2.921	1.223	7.92E-21	1.41E-18
CPA4	1.745	5.018	1.524	7.68E-15	1.77E-13
IFITM10	16.007	7.255	-1.142	2.28E-07	9.62E-07
MXRA8	12.807	32.367	1.338	8.08E-12	8.62E-11
UCHL1	7.112	22.597	1.668	3.43E-15	8.70E-14
SULF2	10.257	27.297	1.412	2.10E-19	2.09E-17
SLC16A1	6.792	17.341	1.352	3.16E-19	2.86E-17
SYNDIG1	0.522	1.504	1.525	8.97E-15	2.02E-13
DIO3	0.525	2.184	2.056	0.000185372	0.000436687
MTATP8P1	1.975	0.863	-1.194	4.31E-12	4.91E-11
SLITRK6	25.133	11.219	-1.164	4.06E-11	3.73E-10
SYT8	49.358	16.088	-1.617	7.73E-08	3.57E-07
MIR4728	2.626	1.202	-1.128	1.14E-11	1.19E-10
MRGPRF	3.038	6.643	1.129	7.58E-08	3.51E-07
MTND4P24	3.201	0.724	-2.144	5.89E-08	2.78E-07
MT1L	3.596	10.498	1.546	2.50E-17	1.18E-15
ALPP	3.569	7.346	1.042	0.020237357	0.030907359
AP001992.1	1.935	0.911	-1.086	2.60E-11	2.50E-10
LTO1	6.968	2.86	-1.285	5.37E-07	2.10E-06
MRC2	10.691	23.523	1.138	1.05E-18	7.91E-17
TREM1	0.903	2.611	1.531	3.89E-15	9.63E-14
GDPD2	5.012	2.165	-1.211	7.71E-07	2.91E-06
HNF1B	4.079	1.869	-1.126	1.29E-13	2.12E-12

MT-ND4L	3470.506	1677.731	-1.049	4.07E-13	5.98E-12
F13A1	3.28	10.876	1.729	4.88E-11	4.39E-10
PLCD3	22.926	10.098	-1.183	1.60E-11	1.59E-10
APCDD1L	0.302	2.781	3.201	1.49E-23	1.21E-20
IPO5P1	6.286	2.416	-1.379	7.43E-23	3.51E-20
CACNA2D1	0.543	1.572	1.533	1.47E-13	2.38E-12
TM4SF1	33.58	70.947	1.079	5.37E-13	7.67E-12
ACSM6	1.96	0.497	-1.98	2.41E-18	1.64E-16
FOXQ1	61.805	23.812	-1.376	3.86E-16	1.32E-14
GJA5	2.091	5.093	1.284	1.14E-06	4.17E-06
SPIRE2	6.171	2.639	-1.226	7.66E-15	1.77E-13
LGALS1	176.654	403.228	1.191	3.39E-18	2.15E-16
ARL14	12.632	3.522	-1.843	3.97E-10	3.00E-09
OXCT1	4.199	9.142	1.122	2.25E-14	4.40E-13
ZNF432	7.394	2.729	-1.438	3.49E-10	2.69E-09
PSCA	1240.084	351.56	-1.819	2.13E-16	7.87E-15
SLC16A2	1.623	3.428	1.078	3.90E-14	7.13E-13
YJEFN3	3.188	1.341	-1.249	1.20E-14	2.60E-13
WSCD2	1.95	0.877	-1.152	2.43E-11	2.34E-10
ABCA12	0.672	1.601	1.253	9.54E-07	3.53E-06
TPSB2	3.862	7.93	1.038	4.60E-06	1.50E-05
TMCC2	0.638	1.402	1.137	9.16E-09	5.10E-08
HOXB5	6.523	2.557	-1.351	1.94E-12	2.43E-11
TPST1	5.797	11.844	1.031	1.10E-22	4.61E-20
LINC02253	1.486	0.729	-1.026	2.14E-05	6.10E-05
AL031058.1	1.861	4.434	1.253	3.93E-12	4.51E-11
SSH3	68.731	33.122	-1.053	4.35E-20	5.34E-18
FAM3D	13.943	6.066	-1.201	8.86E-12	9.37E-11
LINC01341	2.208	0.827	-1.418	9.53E-17	3.93E-15
CDH23	1.689	0.793	-1.091	8.17E-10	5.78E-09
LGALS7B	2.471	25.264	3.354	6.11E-07	2.36E-06
MT1M	1.705	5.421	1.669	5.43E-14	9.69E-13

RGS4	0.822	1.829	1.153	2.19E-15	5.86E-14
SPON1	3.875	8.586	1.148	2.69E-09	1.70E-08
STAB1	4.686	9.692	1.049	7.54E-08	3.49E-07
AC007998.4	2.353	1.064	-1.145	5.03E-14	9.05E-13
CRYAB	4.43	10.265	1.212	8.26E-15	1.88E-13
LINC02154	0.622	5.569	3.163	0.000183643	0.000433162
ADAMTS12	0.856	2.672	1.643	5.20E-22	1.61E-19
PDLIM4	4.706	9.542	1.02	2.63E-09	1.67E-08
MT1E	19.644	41.4	1.076	7.94E-10	5.63E-09
LOXL2	6.109	12.432	1.025	2.71E-14	5.18E-13
CHGA	0.5	3.114	2.64	2.40E-06	8.20E-06
ASMTL-AS1	5.404	2.495	-1.115	2.98E-13	4.49E-12
TMEM238L	6.418	2.745	-1.225	1.96E-09	1.27E-08
CRH	56.128	13.551	-2.05	1.16E-09	8.00E-09
KRT6A	159.756	695.621	2.122	9.74E-09	5.40E-08
SPTSSB	34.148	14.445	-1.241	8.32E-11	7.10E-10
CCL11	3.965	8.338	1.072	1.66E-09	1.09E-08
STX2	1.579	3.255	1.044	1.91E-19	1.93E-17
CD248	11.096	24.308	1.131	1.62E-11	1.61E-10
EGFR	8.347	26.818	1.684	3.06E-13	4.59E-12
ID1	317.029	144.676	-1.132	5.48E-15	1.30E-13
CYP4F29P	4.342	0.962	-2.175	5.12E-13	7.33E-12
FIBIN	1.894	5.327	1.492	1.42E-15	4.05E-14
CGB5	0.246	7.331	4.896	0.004645683	0.008152982
MT2A	124.393	269.069	1.113	2.35E-18	1.62E-16
AC006435.2	1.595	0.771	-1.049	3.34E-15	8.49E-14
SRPX	3.406	13.057	1.939	1.00E-17	5.40E-16
ECM1	8.756	18.399	1.071	4.76E-20	5.68E-18
AL135999.3	4.772	1.97	-1.277	2.34E-12	2.87E-11
SACS	0.613	1.424	1.215	4.70E-16	1.55E-14
TRHDE-AS1	1.95	0.815	-1.259	2.49E-08	1.27E-07
BNC1	1.1	4.484	2.027	3.76E-09	2.29E-08

KLF15	2.528	1.164	-1.119	4.21E-10	3.16E-09
SDR9C7	0.355	1.798	2.339	0.008734248	0.014448948
AC018665.1	4.2	1.76	-1.255	2.48E-17	1.18E-15
KRTDAP	16.265	81.136	2.319	0.004294306	0.007590615
HPGD	81.635	22.889	-1.835	5.55E-16	1.78E-14
RHCG	8.131	36.659	2.173	0.000124457	0.000303409
POPDC3	1.12	2.29	1.032	6.90E-09	3.95E-08
AL354919.2	3.386	0.845	-2.003	1.69E-09	1.11E-08
SFRP4	10.935	28.559	1.385	9.59E-12	1.01E-10
RHPN1	13.987	6.703	-1.061	1.71E-15	4.76E-14
MTCO1P40	24.482	4.657	-2.394	1.06E-08	5.82E-08
FABP6	18.086	5.736	-1.657	8.14E-14	1.40E-12
GPC2	2.452	1.19	-1.043	0.000170277	0.000404127
FBP1	83.074	33.844	-1.296	1.18E-17	6.13E-16
CES1	19.596	42.459	1.116	1.20E-08	6.53E-08
ANXA10	31.206	7.552	-2.047	6.93E-11	6.02E-10
ANGPTL2	8.27	17.719	1.099	7.40E-11	6.40E-10
FN1	55.843	209.454	1.907	1.34E-25	2.49E-22
CDH11	1.985	4.563	1.201	2.70E-13	4.11E-12
ADH1C	11.328	3.016	-1.909	2.47E-05	6.93E-05
TMEM45A	6.362	18.094	1.508	5.55E-16	1.78E-14

EMRGs: energy metabolism‑related genes, FC: fold-change, FDR: false discovery rate.
